# Engineering Curcumin Biosynthesis in Poplar Affects Lignification and Biomass Yield

**DOI:** 10.3389/fpls.2022.943349

**Published:** 2022-07-04

**Authors:** Barbara De Meester, Paula Oyarce, Ruben Vanholme, Rebecca Van Acker, Yukiko Tsuji, Thijs Vangeel, Sander Van den Bosch, Jan Van Doorsselaere, Bert Sels, John Ralph, Wout Boerjan

**Affiliations:** ^1^Department of Plant Biotechnology and Bioinformatics, Ghent University, Ghent, Belgium; ^2^VIB Center for Plant Systems Biology, Ghent, Belgium; ^3^Department of Biochemistry, University of Wisconsin, Madison, WI, United States; ^4^US Department of Energy, Great Lakes Bioenergy Research Center, Wisconsin Energy Institute, Madison, WI, United States; ^5^Center for Sustainable Catalysis and Engineering, KU Leuven, Leuven, Belgium; ^6^VIVES, Roeselare, Belgium

**Keywords:** lignin, lignin engineering, alternative lignin monomers, poplar, curcumin, translational research

## Abstract

Lignocellulosic biomass is recalcitrant toward deconstruction into simple sugars mainly due to the presence of lignin. By engineering plants to partially replace traditional lignin monomers with alternative ones, lignin degradability and extractability can be enhanced. Previously, the alternative monomer curcumin has been successfully produced and incorporated into lignified cell walls of Arabidopsis by the heterologous expression of *DIKETIDE-CoA SYNTHASE* (*DCS*) and *CURCUMIN SYNTHASE2* (*CURS2*). The resulting transgenic plants did not suffer from yield penalties and had an increased saccharification yield after alkaline pretreatment. Here, we translated this strategy into the bio-energy crop poplar. Via the heterologous expression of *DCS* and *CURS2* under the control of the secondary cell wall *CELLULOSE SYNTHASE A8-B* promoter (*ProCesA8-B*), curcumin was also produced and incorporated into the lignified cell walls of poplar. *ProCesA8-B:DCS_CURS2* transgenic poplars, however, suffered from shoot-tip necrosis and yield penalties. Compared to that of the wild-type (WT), the wood of transgenic poplars had 21% less cellulose, 28% more matrix polysaccharides, 23% more lignin and a significantly altered lignin composition. More specifically, *ProCesA8-B:DCS_CURS2* lignin had a reduced syringyl/guaiacyl unit (S/G) ratio, an increased frequency of *p*-hydroxyphenyl (H) units, a decreased frequency of *p*-hydroxybenzoates and a higher fraction of phenylcoumaran units. Without, or with alkaline or hot water pretreatment, the saccharification efficiency of the transgenic lines was equal to that of the WT. These differences in (growth) phenotype illustrate that translational research in crops is essential to assess the value of an engineering strategy for applications. Further fine-tuning of this research strategy (e.g., by using more specific promoters or by translating this strategy to other crops such as maize) might lead to transgenic bio-energy crops with cell walls more amenable to deconstruction without settling in yield.

## Introduction

Lignocellulosic biomass, which is largely composed of plant cell walls, is a promising renewable feedstock for the production of biofuels and bio-based materials (Vanholme et al., 2013a). The polysaccharides present in the plant cell wall, cellulose and hemicelluloses, can be hydrolyzed into fermentable primary sugars by a process called saccharification. These monomers can be further processed into bulk chemicals, such as bioethanol, levulinic acid, and 2,5-furandicarboxylic acid (Bozell et al., 2007; Corma et al., 2007; Vanholme et al., 2013a; Isikgor and Becer, 2015; de Vries et al., 2021b). However, the saccharification process is hindered by the presence of lignin. This complex aromatic heteropolymer is crucial to plant growth and development; it provides rigidity to plant cells, provides imperviousness to water conducting cells, and plays a role in defense to pathogens. The lignin polymer is mainly derived from the monolignols coniferyl and sinapyl alcohol and low levels of *p*-coumaryl alcohol. After polymerization in the cell wall, the monolignols produce guaiacyl (G), syringyl (S), and *p*-hydroxyphenyl (H) units, respectively (Vanholme et al., 2019). To make the polysaccharides accessible to the hydrolytic enzymes, however, lignocellulosic biomass needs to be pretreated with heat and/or (physico)chemical methods. These pretreatments are costly and energy-demanding. There is therefore interest in engineering lignin of biomass crops to facilitate cell wall deconstruction (Sederoff et al., 1999; Boerjan et al., 2003; Ralph et al., 2004b,^2019^; Chen and Dixon, 2007; Vanholme et al., 2012; Mottiar et al., 2016; Chanoca et al., 2019; Halpin, 2019; Mahon and Mansfield, 2019; De Meester et al., 2020).

Studies of different naturally occurring or transgenic and mutant plants have led to the discovery that plants can tolerate large shifts in lignin composition, often without visible effects on plant development and morphology (Meyer et al., 1998; Marita et al., 1999; Franke et al., 2000; Stewart et al., 2009; Eudes et al., 2017; de Vries et al., 2018). This notion has steered research toward the biosynthesis and incorporation of alternative monomers into the lignin (Grabber et al., 2008, 2010, 2019; Vanholme et al., 2012; Smith et al., 2015, 2022; Mottiar et al., 2016; del Río et al., 2022). Enhancing the incorporation of atypical monomers that are rare, or even absent, in the lignin of wild-type (WT) plants could permit a more efficient conversion of lignocellulosic biomass for the industry while maintaining the biological role of lignin. Over the past years, several studies have evaluated the incorporation of alternative monomers into the lignin polymer of several plant species. For example, overexpression of the gene encoding the enzyme FERULOYL-CoA MONOLIGNOL TRANSFERASE in poplar has led to higher amounts of monolignol ferulates incorporated into the lignin, which considerably increased the saccharification efficiency and chemical pulping (Wilkerson et al., 2014; Kim et al., 2017; Zhou et al., 2017). Similarly, (enhanced) *p*-coumaroylation of Arabidopsis and poplar lignins was achieved by the expression of *p-COUMAROYL-CoA MONOLIGNOL TRANSFERASES* from *Brachypodium distachyon* or *Oryza sativa*, resulting in an increased saccharification efficiency without compromising the plant phenotype (Smith et al., 2015; Sibout et al., 2016; Lapierre et al., 2021). Expression of a bacterial *HYDROXYCINNAMOYL-COA HYDRATASE-LYASE* (*HCHL*) in Arabidopsis resulted in the incorporation of *p*-hydroxybenzaldehyde and *p*-hydroxybenzoate into the lignin (Eudes et al., 2012). The resulting HCHL-engineered plants had a lignin polymer with a reduced molecular weight; biomass from these plants was more easily saccharified after pretreatment. The combinatorial downregulation of *COMT* and overexpression of *F5H* in Arabidopsis led to more than 30% of easily cleavable benzodioxane linkages in the lignin due to an increased incorporation of 5-hydroxyconiferyl alcohol (Vanholme et al., 2010; Weng et al., 2010). Disrupting *FLAVONE SYNTHASE II* (*FNSII*) in rice resulted in an altered cell wall, incorporating the intermediate flavanone naringenin, instead of tricin, into the lignin polymer (Lam et al., 2017). The *fnsII* mutant plants showed a normal growth and an enhanced saccharification efficiency. The incorporation of naringenin into poplar lignin was achieved by expressing an apple *CHALCONE SYNTHASE* 3 (Mahon et al., 2022). Similarly to the engineered rice plants, biomass from the transgenic trees had increased saccharification yields from plants that grew normally. Finally, heterologous expression of a gene encoding a bacterial 3-DEHYDROSHIKIMATE DEHYDRATASE in hybrid poplar resulted in reduced lignin amounts, the incorporation of monolignol-3,4-dihydroxybenzoate conjugates in the lignin, and improved saccharification without affecting growth (Unda et al., 2022).

**FIGURE 1:**
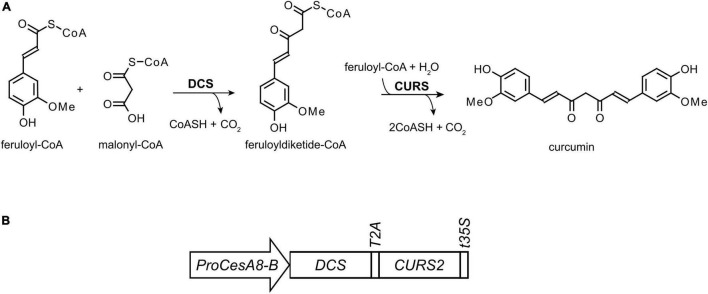
Curcumin biosynthesis. **(A)** In turmeric, DIKETIDE-CoA SYNTHASE (DCS) and CURCUMIN SYNTHASE (CURS) convert two molecules of feruloyl-CoA and one molecule of malonyl-CoA into curcumin, which is characterized by two phenolic ring structures linked via a 7-carbon unsaturated aliphatic chain. **(B)** For the biosynthesis of curcumin in poplar, the coding sequences of *DCS* and *CURS2* genes were linked together by the *T2A* sequence, and put under control of the *CELLULOSE SYNTHASE 8-B* promoter (*ProCesA8-B*) and terminated with a *35S* terminator sequence (*t35S*).

We previously engineered Arabidopsis to incorporate the alternative monomer curcumin into the lignin polymer (Oyarce et al., 2019). Curcumin, a metabolite naturally occurring in *Curcuma longa* (turmeric), has two phenolic rings linked by a seven carbon chain containing two conjugated carbonyl functionalities (Figure 1A; Chen and Tan, 1998). The conjugated 9-carbonyl function at the *para*-position of the two phenolic hydroxyl functions eases the cleavage of 8-*O*-4 inter-unit linkages under alkaline conditions (Oyarce et al., 2019). More specifically, the required hydrolysis temperature of the 8-*O*-4 linkage can be lowered from ∼170°C (for traditional lignin β-ether units) to ∼65°C (Criss et al., 1998; Imai et al., 2007; Tsuji et al., 2015; Mnich et al., 2017). In addition, the β-diketone functionality in the aliphatic chain of curcumin can be cleaved under alkaline conditions at temperatures as low as 30°C (Hauser et al., 1948; Pearson and Mayerle, 1951; Tønnesen and Karlsen, 1985; Price and Buescher, 1997; Wang et al., 1997; Tomren et al., 2007). In transgenic Arabidopsis, curcumin biosynthesis was achieved by the expression of the genes encoding the enzymes DIKETIDE-CoA SYNTHASE (DCS) and CURCUMIN SYNTHASE 2 (CURS2), that catalyze the conversion of one malonyl-CoA and two feruloyl-CoA molecules into curcumin (Figure 1A; Katsuyama et al., 2009; Oyarce et al., 2019). Using the secondary cell wall *CELLULOSE SYNTHASE A4* promoter (*ProCesA4*), the expression of the biosynthetic genes was restricted to lignified cells. The *ProCesA4:DCS_CURS2* Arabidopsis lines were successful at incorporating curcumin into the lignified cell wall without affecting the plant’s biomass yield. Moreover, the transgenic Arabidopsis lines had an increased saccharification efficiency after alkaline pretreatment. In this work, we investigated the translation of this strategy into the bio-energy crop poplar.

## Materials and Methods

### Plant Material and Growth Conditions

The creation of the vector *pDONR221-DCS_CURS2* is described in Oyarce et al. (2019). The *CELLULOSE SYNTHASE 8-B* promoter (*ProCesA8-B*) from *P. trichocarpa* (Song et al., 2010) was synthesized by Genscript (Piscataway, NJ, United States) in a pUC57-Kan vector. Sequence information can be found in Supplementary Table 1. The expression vector *ProCesA8-B:DCS_CURS2* was created by an LR multisite II reaction with donor vectors *pUC57-ProCesA8B* and *pDONR221-DCS_CURS2* and destination vector *pALS7m24GW*. After confirmation by PCR, the expression vector was introduced into *Agrobacterium tumefaciens* strain C58C1 PMP90 by electroporation and positive colonies harboring the construct were selected by PCR. Agrobacterium-mediated transformation of *Populus tremula* × *P. alba* 717-B4 was performed according to Brasileiro et al. (1992).

In total, 28 independent transgenic lines (one individual plant for each line) and WT controls (eight plants) were transferred from *in vitro* culture to soil in pots of 5.5 cm diameter, placed in a tray filled with water and covered with a cage liner (Tecniplast APET disposable cage liner for cage body 1291H) for acclimatization. After 2 weeks, one side of the cage liner was lifted and kept accordingly for 1 day, after which the other side was also lifted, to gradually reduce humidity. After 2 weeks of growing in the greenhouse (16 h light/8 h dark photoperiod at ± 21°C), the plants were transferred to 10-L pots filled with a Saniflor^®^ 691 commercial soil (Van Israel). After growing for 87 days in the greenhouse (first growth cycle), the *ProCesA8-B:DCS_CURS2* and WT poplars were harvested for further analysis. After harvesting, new shoots developed from the stool. After growing for another 4 months in the greenhouse (second growth cycle), the *ProCesA8-B:DCS_CURS2* and WT poplars were harvested for bright-field microscopy.

### Harvesting of Plant Material

After 87 days of growth in soil in the greenhouse (first growth cycle), the WTs reached heights of approximately 130 cm. At this time point, all poplars were harvested by cutting the stem 10 cm above the base leaving 2–3 axillary buds to allow development of new shoots. The lower 10–15 cm part was debarked and stored in ethanol until future use for fluorescence microscopy. Subsequently, the top 20 cm of the stem was removed. The 6-cm stem part below the removed 20 cm top was debarked, frozen in liquid nitrogen, cut in about 30 pieces and stored at –70°C until further use for phenolic profiling. The leftover stem piece was debarked, air-dried and ground in a ball mill for catalytic hydrogenolysis, cell wall analysis and saccharification.

After 4 months of growth in soil in the greenhouse (second growth cycle), the WTs reached heights of approximately 2 m. At this time point, the poplars were harvested by cutting the main stem 10 cm above the base. The lower 10- to 15-cm part was debarked, stored in tap water and imaged by bright-field microscopy the same day.

### Phenolic Profiling

Soluble phenolic compounds were extracted from approximately 100 mg of stem material (about 6 to 10 pieces of stem from the first growth cycle; see above) with 1 mL methanol at 70°C for 15 min under 1,000 rpm shaking. After centrifugation at room temperature and maximum speed, 800 μL of the supernatant was dried under vacuum and the pellet was resuspended in 100 μL of cyclohexane and 100 μL of 1/1 DMSO/water (v/v). The tubes were vortexed and centrifuged at 14,000 rpm (20,000 x g) for 10 min, after which a 15-μL aliquot of the aqueous phase was injected on a Ultra-High-Performance Liquid Chromatography (UHPLC) system (Waters Acquity UPLC^®^) equipped with a BEH C18 column (2.1 × 150 mm, 1.7 μM, Waters) and hyphenated to a time-of-flight mass spectrometer (TOF MS, Synapt Q-Tof, Waters Corporation, Milford, Massachusetts, United States), using gradient elution. Gradient elution information, negative-ion mode mass spectrometry setting, chromatogram integration and alignment via Progenesis QI software (Waters) were performed as previously described (Eloy et al., 2017). Peak abundances were normalized to the dry weight (mg) of the pellet remaining after methanol extraction and drying on a SpeedVac. Principal Component Analysis (PCA) was performed in R software via the “prcomp” command.

### Microscopy

For fluorescence microscopy, the stem piece (see above) from the first growth cycle was incubated for three days in 70% ethanol. Slices of 15 μm thick were made using a Reichert-Jung 2040 Autocut Microtome (Leica). Next, the slices were incubated for (i) 2 h in tap water or (ii) 2 h in acetone followed by 2 h in tap water to wash away the soluble compounds. The fluorescence of curcumin and lignin was visualized using the Zeiss LSM 780 microscope with an iLCI Plan-Neofluar 25x/0.8 Imm Korr DIC M27 objective (0.6 zoom). The fluorescence signal of curcumin was obtained at an excitation wavelength of 488 nm and emission wavelength between 490 and 578 nm. For lignin visualization, an excitation wavelength of 350 nm and emission wavelength between 407 and 479 nm was used.

For bright-field microscopy, the stem piece (see above) from the second growth cycle was cut into slices of 15 μm thick using a Reichert-Jung 2040 Autocut Microtome (Leica). Next, the slices were incubated for 2 h in acetone followed by 2 h in tap water. The sections were imaged with an Olympus BX51 microscope (Olympus) with an Olympus PlanC N 10x (0.25 NA) objective.

### Catalytic Hydrogenolysis

Ground wood powder (see above) from the first growth cycle was used to prepare Cell Wall Residue (CWR) by sequentially washing the wood powder for 30 min each with milliQ water at 98°C, ethanol at 76°C, chloroform at 59°C and acetone at 54°C. Three hundred mg of CWR was incubated with 40 mg (5 wt% Pd) Pd/C catalyst and 40 mL methanol in a 100-mL Parr batch reactor at 250°C for 3 h with an initial H_2_-pressure of 20 bar (at room temperature). After this reaction, the reactor was cooled and washed with acetone to collect all products. The reaction mixture was filtered and the filtrate was evaporated using a rotary evaporator. A part (roughly two thirds) of this product mixture was further separated into carbohydrate products and lignin products via liquid/liquid extraction: 2.5 mL of water was added and subsequently threefold extracted with 1 mL of dichloromethane (DCM). The DCM phases were combined and the DCM was evaporated using a N_2_ flow, followed by oven drying at 80°C overnight, yielding a brown, viscous lignin oil. The resulting lignin oil samples were transferred quantitatively in 10-mL crimp cap vials, extracted in 150 μL methanol/150 μL milliQ water and centrifuged for 15 min at 19,757 × *g*. The supernatant (100 μL) was analyzed via UHPLC–MS following the parameters described by Vanholme et al. (2013b) using a gradient of two buffers [buffer A (99/1/0.1 H_2_O/ACN/formic acid pH3), buffer B (99/1/0.1 ACN/H_2_O/formic acid pH3)], but with the following modification: between 0 to 30 min from 95% A and 5% B to 50% A and 50% B, between 30 to 40 min from 50% A and 50% B to 0% A and 100% B. Chromatograms were visualized in Masslynx V4.1 software (Waters) and peaks were integrated using the default parameters (using automatic noise measurement and 2x smoothing).

### Cell Wall Analysis

To determine the cellulose, matrix polysaccharide and lignin amount, ground wood powder (see above) from the first growth cycle was used for preparing CWR by sequentially washing for 30 min each with milliQ water at 98°C, ethanol at 76°C, chloroform at 59°C and acetone at 54°C. The remaining CWR was dried under vacuum and was determined gravimetrically (expressed as mass percentage of dry weight). To determine the crystalline cellulose level, the Updegraff method was used on 10 mg of CWR essentially as described by Updegraff (1969) and modified according to De Meester et al. (2020). The mass loss upon trifluoroacetic acid digestion was used to determine the matrix polysaccharide content (including mainly hemicelluloses, but also pectins and amorphous cellulose). Lignin content was determined by the acetyl bromide method on 5 mg of CWR essentially as described by Dence (1992) and modified according to Van Acker et al. (2013).

Lignin composition was determined by two-dimensional Heteronuclear Single-Quantum Coherence Nuclear Magnetic Resonance (2D HSQC NMR). Two hundred and fifty mg of ground wood powder (see above) from the first growth cycle was extracted three times with distilled water, three times with 80% aqueous ethanol, and once with acetone. The extracted samples were ball-milled using a Fritsch Planetary micro mill Pulverisette 7 vibrating at 600 rpm with zirconium dioxide (ZrO_2_) vessels containing ZrO_2_ ball bearings (10 mm x 10). One cycle of the ball-milling condition consists of 5 min milling and 5 min cooling cycle, and cycle numbers were dependent on each amount of sample. Samples were digested (72 h x 2) with Cellulysin^®^ Cellulase, *Trichoderma viridae* (Calbiochem), at 35°C in acetate buffer (pH 5.0) in order to obtain the Enzyme Lignin (EL). ELs containing small amounts of residual polysaccharides were dissolved into DMSO-d_6_/pyridine-d_5_ (4:1) and subjected to NMR using a Bruker Biospin NEO 700-MHz spectrometer fitted with a cryogenically-cooled QCI ^1^H/^31^P/^13^C/^15^N gradient cryoprobe with inverse geometry (proton coil closest to the sample). 2D-^1^H–^13^C HSQC spectra were acquired using Bruker’s pulse program (hsqcetgpsip2.2). Bruker’s Topspin 3.2 (Mac) software was used to process spectra. The central DMSO peak was used as internal reference (δ_C_: 39.50, δ_H_: 2.49 ppm).

### Saccharification Assays

Saccharification assays were performed on 10 mg of ground wood powder (see above) from the first growth cycle as described by Van Acker et al. (2016). The activity of the 10× diluted enzyme mix was 0.14 Filter Paper Units/mL. For the alkali pretreatment, the stem material was treated with 1 mL 0.25% (v/v) NaOH at 90°C for 3 h while shaking at 750 rpm. For the hot water pretreatment, the stem material was incubated for 3 h with water at 90°C. The cellulose-to-glucose conversion was calculated based on the amount of glucose released upon saccharification and the original cellulose content that was measured for each sample.

## Results

### *ProCesA8-B:DCS_CURS2* Poplars Produce Curcumin and Curcumin-Derived Metabolites

To produce curcumin in the lignified tissues of poplar (*Populus tremula x P. alba*), the curcumin biosynthetic genes *DCS* and *CURS2*, linked together by a sequence coding for the self-cleaving T2A peptide, were expressed under the control of the *P. trichocarpa* secondary cell wall *CesA8-B 8-B* promoter (Figure 1B). This promoter was previously shown to be successful for lignin engineering in poplar (Wilkerson et al., 2014) and confers high expression in developing xylem cells (Joshi et al., 2004; Suzuki et al., 2006; Song et al., 2010). In total, 28 independent *ProCesA8-B:DCS_CURS2* poplar lines were obtained after Agrobacterium-mediated transformation and selection. After transfer to soil, all 28 transgenic lines and their WT controls were grown for 87 days in the greenhouse, after which they were harvested for further analysis.

To investigate whether the expression of *DCS* and *CURS2* resulted in the production of the envisioned compounds, phenolic metabolites were extracted from 87-day-old poplar xylem, and subsequently analyzed via UHPLC-MS. Upon a targeted search, five “curcuminoids” were found to be produced in all 28 transgenic poplars, whereas these compounds were absent in the WT (Table 1 and Supplementary Figure 1). In addition to free curcumin (**1**), different coupling products between curcumin and coniferyl alcohol were identified: two isomers of curcumin(4-*O*-8)G (**2**–**3**), curcumin(5–8)G (**4**) and curcumin(8-8)G (**5**), reflecting the three main interunit bonds that result from radical coupling of curcumin with coniferyl alcohol.

**TABLE 1:** Targeted analysis of phenolic metabolites in *ProCesA8-B:DCS_CURS2* poplar stems.

No.	Compound name	*m/z*	R.T. (min)	WT	*ProCesA8-B:DCS_CURS2*	Ratio
				mean ± SD	mean ± SD	
**Curcuminoids**						
1	Curcumin (enol)^1^	367.120	26.55	n.d.	2307 ± 1308**	∞
2	Curcumin(4-*O*-8)G 1^1^	563.193	23.86	n.d.	4846 ± 1899**	∞
3	Curcumin(4-*O*-8)G 2^1^	563.193	27.60	n.d.	1328 ± 991**	∞
4	Curcumin(5-8)G^2^	545.184	27.63	n.d.	909 ± 732**	∞
5	Curcumin(8-8)G^2^	545.183	25.17	n.d.	1290 ± 666**	∞
**Phenylpentanoid monomers**						
6	Dihydroferuloyl-β-keto acid^1^	237.076	6.22	208 ± 118	16451 ± 23292**	79
7	Dihydroferuloyl-β-keto acid + glycerol^2^	311.112	4.80	n.d.	1005 ± 817**	∞
8	Dihydroferuloyl-β-keto acid + malate^2^	353.087	6.93	n.d.	626 ± 460**	∞
9	Dihydroferuloyl-β-keto acid + hexose^1^	399.130	3.09	1990 ± 1229	103329 ± 70530**	52
10	Dihydroferuloyl-β-keto acid + 302 Da^1^	539.177	10.26	n.d.	31468 ± 19507**	∞
11	Tetrahydroferuloyl-β-keto acid + hexose^2^	401.145	4.13	n.d.	4177 ± 4544**	∞
**Phenylpentanoid-containing dimers**						
12	Dihydroferuloyl-β-keto acid(4-*O*-8)G^1^	433.142	7.78	216 ± 139	12467 ± 10109**	58
13	Dihydroferuloyl-β-keto acid coniferyl alcohol cyclobutane dimer^2^	417.157	12.47	282 ± 155	3536 ± 2048**	13
14	[Dihydroferuloyl-β-keto acid(8-5)G or G(8-5)dihydroferuloyl-β-keto acid] + hexose^2^	577.192	8.30	n.d.	606 ± 526**	∞

When compared to WT, the abundance of curcuminoids and phenylpentanoids is increased in the transgenic lines (Student’s t-test; **P < 0.01; WT, n = 8 biologically independent replicates; ProCesA8-B:DCS_CURS2, n = 28 biologically independent lines). Peak area (mean) ± standard deviation (SD) are expressed in counts. R.T., retention time; G, guaiacyl unit. Remark: signals below 100 counts are considered as not detected (n.d.). ^1^Tentatively structurally characterized based on MS/MS spectral analysis for ProCesA8-B:DCS_CURS2 and based on m/z and R.T. for WT. ^2^Annotated based on m/z and R.T. similarity with structurally characterized compounds in Oyarce et al. (2019) for both ProCesA8-B:DCS_CURS2 and WT.

In addition to curcumin-derived compounds, *ProCesA8-B:DCS_CURS2* poplars also had an increased abundance of derivatives of the phenylpentanoid intermediate (Table 1 and Supplementary Figure 1). More specifically, six phenylpentanoid monomers, defined as compounds in which the phenylpentanoid structure was free or linked either via a 4-*O*-ether or γ-*O*-ester to a non-phenolic moiety, were found: dihydroferuloyl-β-keto acid was characterized as a free compound (**6**), or coupled to glycerol (**7**), malate (**8**), hexose (**9**), or a subunit of unknown identity (**10**). Additionally, tetrahydroferuloyl-β-keto acid coupled to hexose was characterized (**11**). Three phenylpentanoid-containing dimers, that are defined as compounds in which a phenylpentanoid moiety was linked via 4-*O*-8, 8-8, 8-5 or a cyclobutane structure to a phenylpropanoid or a second phenylpentanoid moiety, were also characterized: dihydroferuloyl-β-keto acid coupled with a 4-*O*-8 bond or a cyclobutane structure to coniferyl alcohol (**12**–**13**), and an 8-5 coupling product of dihydroferuloyl-β-keto acid and coniferyl alcohol with a hexose (**14**).

### Curcumin Is Incorporated Into the Secondary Cell Wall of *ProCesA8-B:DCS_CURS2* Lines

**FIGURE 2:**
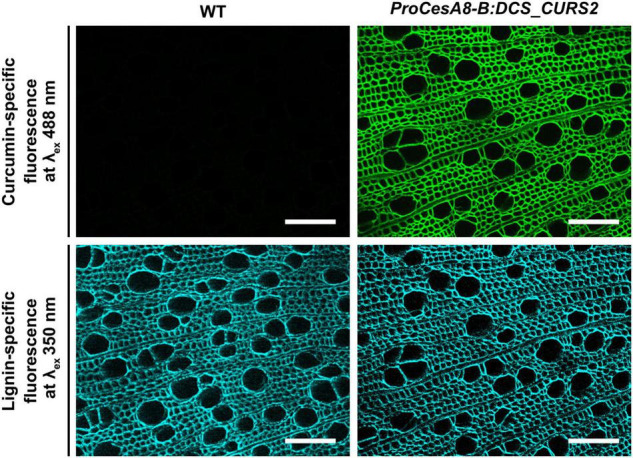
Fluorescence microscopy on transverse stem sections of *ProCesA8-B:DCS_CURS2* poplars. The curcumin-specific fluorescence signal (excitation wavelength of 488 nm) was observed in the cell wall of *ProCesA8-B:DCS_CURS2* poplars, but not in that of WT poplars. The lignin-specific fluorescence (excitation wavelength of 350 nm) was observed in both the WT and *ProCesA8-B:DCS_CURS2* lines. Scale bars: 100 μm.

Curcumin needs to be incorporated into the lignin polymer before it can render the lignin polymer more susceptible to alkaline pretreatments. Curcumin is fluorescent at an excitation wavelength of 488 nm, a spectral property that was successfully used to visualize the localization of curcumin in the cell wall region of inflorescence stem cross-sections of *ProCesA4:DCS_CURS2* Arabidopsis (Oyarce et al., 2019). To investigate the localization of curcumin in 87-day-old *ProCesA8-B:DCS_CURS2* poplars, cross-sections of the stem were examined via fluorescence microscopy. The curcumin-specific fluorescence signal was present in the cell walls of *ProCesA8-B:DCS_CURS2* xylem cells, but was absent in those of WT cells (Figure 2). The curcumin-specific fluorescence signal remained after washing the cross sections in acetone to remove soluble metabolites (Supplementary Figure 2), establishing that curcumin was tightly linked to the secondary cell wall. Additionally, lignin autofluorescence was used to visualize the architecture of the vessels in WT and *ProCesA8-B:DCS_CURS2* stems. The *ProCesA8-B:DCS_CURS2* xylem was similar to that of the WT and displayed no vascular defects (Figure 2).

Because the NMR signals from curcumin and G-lignin units overlap (Oyarce et al., 2019), this technique could not be used to confirm that curcumin was incorporated into the lignified cell wall of the (Arabidopsis and poplar) transgenic lines. Therefore, similar as for *ProCesA4:DCS_CURS2* Arabidopsis, catalytic hydrogenolysis was performed on extract-free cell wall material derived from the dried, debarked stems of 87-day-old *ProCesA8-B:DCS_CURS2* poplars to confirm curcumin incorporation into their cell wall. During catalytic hydrogenolysis, cell wall material is processed with a redox catalyst under reductive conditions, resulting in the cleavage of alkyl aryl ether bonds in the lignin, the removal of secondary (benzylic) alcohols, and the reduction of aliphatic double bonds (Galkin and Samec, 2016; Renders et al., 2019). However, aromatic structures and phenolic moieties are not hydrogenated and the cell wall polysaccharides remain largely intact (Van den Bosch et al., 2015). Here, Pd/C was used as a catalyst, retaining the largest fraction of primary alcohols (Li et al., 2018; Abu-Omar et al., 2021). Next, the lignin oil fraction was analyzed via UHPLC-MS. Based on principal component analysis of all detected peaks, *ProCesA8-B:DCS_CURS2* samples separated from WT samples (explaining 25% of the variation in PC1; Supplementary Figure 3A). Subsequently, we performed a targeted search for peaks that were derived from curcumin (coupling products) based on the structurally characterized products found in *ProCesA4:DCS_CURS2* Arabidopsis lignin oils (i.e., tetrahydrocurcumin, hexahydrocurcumin, deoxyhexahydrocurcumin and deoxyoctahydrocurcumin(5-8)G; Oyarce et al. (2019). Two peaks were found for which the intensity was at least tenfold higher in *ProCesA8-B:DCS_CURS2* compared to WT (Supplementary Figures 3B,C). These two peaks matched with hexahydrocurcumin (an incomplete reduction product of curcumin) and with deoxyoctahydrocurcumin(5-8)G, hereby confirming the ability of curcumin to couple with traditional monomers in the cell wall of poplar (Supplementary Figures 3B,C).

In conclusion, fluorescence microscopy and catalytic hydrogenolytic analysis showed that curcumin incorporated into the secondary cell wall of *ProCesA8-B:DCS_CURS2* poplars.

### Engineering the Curcumin Pathway Into Poplar Affects Plant Growth

**FIGURE 3:**
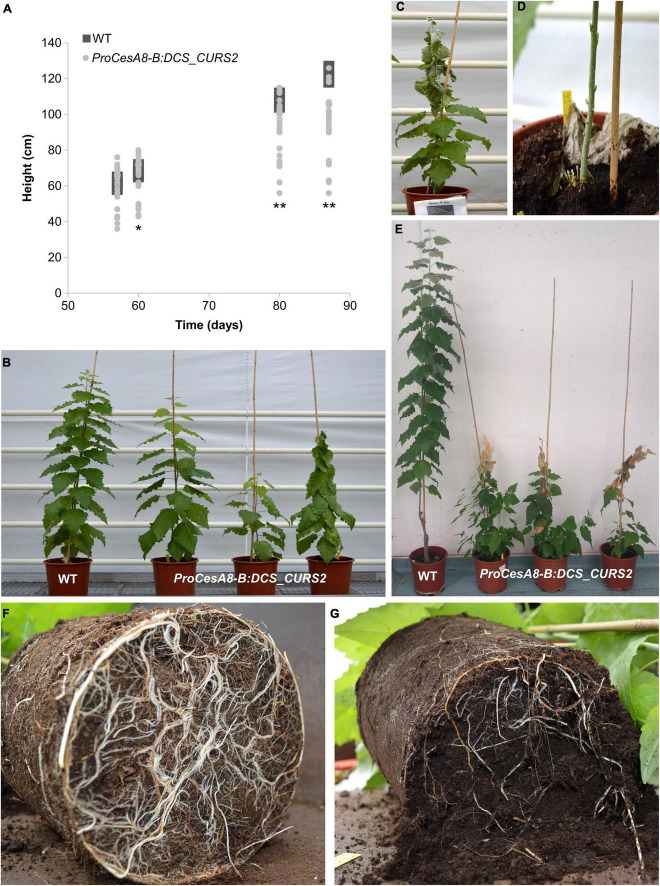
*ProCesA8-B:DCS_CURS2* poplars display growth perturbations. **(A)** Growth curves of WT and *ProCesA8-B:DCS_CURS2* poplars over a period of 87 days. Individual values are represented by squares (WT) or dots (*ProCesA8-B:DCS_CURS2*). Differences in growth between the WT and the transgenic lines were assessed with Student’s *t*-test (*0.01 < *P* < 0.05; **P < 0.01; WT, *n* = 8 biologically independent replicates; *ProCesA8-B:DCS_CUR*S2, *n* = 28 biologically independent lines). **(B)** During the 87-day growth period, a lot of variation was observed in the severity of the growth perturbation of *ProCesA8-B:DCS_CURS2* poplars. **(C,D)** After ceasing growth, the shoot tip of some transgenic poplars started to senesce **(C)**, after which many new secondary shoots emerged from the roots and basal part of the stem **(D)**. **(E)** Image taken after a second 4-month growth period (after pruning the poplars after their first 87-day-long growth period); the WT reached a height of approximately 2 m, at which point the tip of the main stem of the *ProCesA8-B:DCS_CURS2* poplars had become necrotic at a height of approximately 1 m. However, a lot of secondary shoots sprouted from the roots and the basal part of the main stem. **(F,G)** The root system of *ProCesA8-B:DCS_CURS2* poplars **(G)** was underdeveloped compared to that of the WT **(F)** after growing in the greenhouse for 87 days. Scale bars: 20 cm in **(B,C,E)** and 10 cm in **(D,F,G)**.

To determine the influence of the introduction of the curcumin biosynthetic pathway on the development of poplar, the height of WT and *ProCesA8-B:DCS_CURS2* poplars was monitored over their 87-day growth period (Figure 3A). After growing for 57 days in soil, no significant differences in height were observed between WT and *ProCesA8-B:DCS_CURS2* poplars. However, from day 60 onward, the growth rate of the transgenic poplars gradually declined until most of them ceased to grow (Supplementary Table 2). During this growth period, a lot of variation was observed in the height of *ProCesA8-B:DCS_CURS2* poplars (Figures 3A,B and Supplementary Table 2). At the time of harvest on day 87 (first growth cycle), three out of 28 transgenic poplars had shoot tips (several tens of centimeters, depending on the plant) that started to desiccate (Figure 3C), while already developing many new secondary shoots from the basal part of the stem and the roots (Figure 3D). After growing for 87 days in the greenhouse, the root system of the transgenic poplars was underdeveloped when compared to the WT (Figures 3F,G).

After pruning, the poplars were grown again for a second growth cycle of 4 months. In this case, we observed that, after ceasing growth, all 28 *ProCesA8-B:DCS_CURS2* lines had shoot tips that desiccated and became necrotic. As in the first growth cycle, this shoot-tip necrosis was accompanied by the formation of secondary shoots leading to a “bushy” phenotype (Figure 3E). To investigate whether the shoot-tip necrosis of 4-month-old *ProCesA8-B:DCS_CURS2* poplars was correlated with vascular collapse, stem cross sections were visualized using bright-field microscopy (Supplementary Figure 4). The cross-sections were washed with acetone to remove soluble metabolites. Interestingly, the walls of secondary-thickened cells of *ProCesA8-B:DCS_CURS2* transgenic plants showed a yellow coloration, indicative of the presence of curcumin (or a curcumin-derived compound), whereas those of the WT were gray. Both WT and transgenic lines showed round, open vessels.

### *ProCesA8-B:DCS_CURS2* Poplars Have Altered Cell Wall Composition

*ProCesA8-B:DCS_CURS2* poplars displayed yield penalties and produced and incorporated curcumin into their cell wall. To study additional effects on the cell wall, the CWR, the cellulose, matrix polysaccharide and lignin contents and the lignin composition of the basal part of the dried, debarked stem of 87-day-old WT and *ProCesA8-B:DCS_CURS2* poplars were determined (Table 2). Interestingly, compared to the WT, transgenic poplars had ∼10% less CWR, and thus relatively more soluble compounds. The crystalline cellulose content per CWR was decreased from 40.4% in the WT to 32.1% in the *ProCesA8-B:DCS_CURS2* poplars, while the amount of matrix polysaccharides (including hemicelluloses, pectins and amorphous cellulose) in the CWRs was increased from 40.8% in the WT to 52.4% in the *ProCesA8-B:DCS_CURS2* lines. The acetyl bromide lignin content per CWR was increased from 15.8% in the WT to 19.4% in *ProCesA8-B:DCS_CURS2*.

**TABLE 2:** Cell wall characteristics.

	WT	*ProCesA8-B:DCS_CURS2*
CWR (% dry weight)	90.2 ± 2.8	79.5 ± 4.5**
Cellulose (% CWR)	40.4 ± 6.2	32.1 ± 8.0*
Matrix polysaccharides (% CWR)	40.8 ± 3.5	52.4 ± 8.8**
Acetyl bromide lignin (% CWR)	15.8 ± 1.2	19.4 ± 1.0**
**NMR-derived aromatic units**		
% H	0.2 ± 0.1	1.9 ± 0.8**
% S	66.1 ± 1.2	52.2 ± 5.0**
% G	33.8 ± 1.2	45.8 ± 4.6**
S/G	1.96 ± 0.10	1.16 ± 0.23**
% PB	5.9 ± 0.9	0.7 ± 0.9**
**NMR-derived interunit linkages**		
% β-Aryl ether (8-*O*-4; A)	89.1 ± 1.9	87.2 ± 2.1
% Phenylcoumaran (8-5; B)	1.8 ± 1.3	3.8 ± 1.3**
% Resinol (8-8; C)	9.1 ± 0.7	9.0 ± 1.0

**FIGURE 4:**
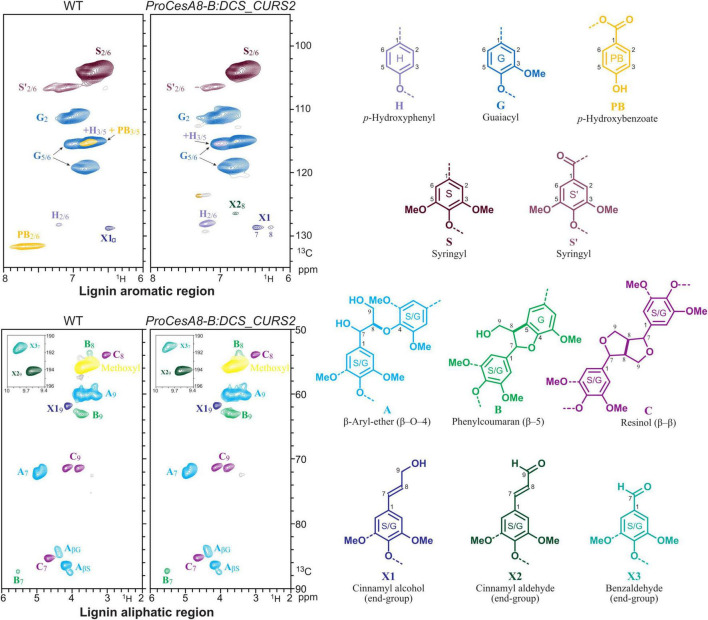
Structural characterization of lignin via NMR. HSQC spectra of the aromatic and oxygenated aliphatic regions of whole cell walls from stems of WT and *ProCesA8-B:DCS_CURS2* poplars. Integrated values for each monomeric unit H, G, S and *p*-hydroxybenzoate and the α-C/H correlation peaks from the major lignin interunit structures A–C are provided in Table 2. The colors of the substructures shown match those of the corresponding signals in the HSQC spectra (where they are resolved). Representative figure for WT, *n* = 8 biologically independent replicates and *ProCesA8-B:DCS_CURS2*, *n* = 28 biologically independent lines.

The cell wall residue (CWR) expressed as mass percentage of dry weight was determined gravimetrically after a sequential extraction. Crystalline cellulose content was determined by the Updegraff method and the mass loss during TFA extraction was used as an estimate of the amount of matrix polysaccharides. Lignin content was determined via the acetyl bromide (AcBr) method and expressed as mass percentage of CWR. Lignin composition was determined via 2D HSQC NMR. Differences between the WT and the transgenic lines were assessed with Student’s t-test (*0.01 < P < 0.05; **P < 0.01; WT, n = 8 biologically independent replicates; ProCesA8-B:DCS_CURS2, n = 28 biologically independent lines). H, p-hydroxyphenyl; S, syringyl; G, guaiacyl; PB, p-hydroxybenzoate (see also Figure 4).

In order to gain further insights into the structural changes caused by curcumin biosynthesis and/or cross-coupling into the lignin polymer, 2D-NMR was performed on lignins from dried, debarked stems of 87-day-old WT and *ProCesA8-B:DCS_CURS2* lines. By analyzing the aromatic and aliphatic regions of the 2D ^1^H–^13^C correlation HSQC spectra, it was possible to visualize differences in lignin monomeric composition and interunit linkages (Figure 4 and Table 2; Mansfield et al., 2012). The fraction of S units was decreased from 66.1% in the WT to 52.2% in the transgenic lines, whereas the fraction of G units was increased from 33.8% in WT to 45.8% in *ProCesA8-B:DCS_CURS2*. Consequently, the S/G ratio (calculated by dividing the proportion of S units by the proportion of G units) was reduced from 1.96 in the WT to 1.16 in *ProCesA8-B:DCS_CURS2*. Notably, the NMR resonance signals originating from the phenolic ring structures of curcumin are not distinguishable from those originating from G units (Oyarce et al., 2019), thus (part of) the increase in G units measured in the transgenic lines could potentially be attributed to the incorporation of curcumin into the lignin polymer. The frequency of H units was increased from 0.2% in the WT to 1.9% in the *ProCesA8-B:DCS_CURS2* lines, whereas the frequency of *p*-hydroxybenzoates in the lignin polymer was reduced from 5.9% in the WT to 0.7% in *ProCesA8-B:DCS_CURS2*. The interunit linkage types were deduced from the oxygenated aliphatic region of the HSQC. In WT, the relative fraction of β-aryl ether (8-*O*-4) linkages and resinol (8-8) linkages was 89.1 and 9.1%, respectively. These fractions did not significantly differ in the *ProCesA8-B:DCS_CURS2* lines. On the other hand, WT contained 1.8% phenylcoumaran (8-5) linkages and this fraction was significantly increased to 3.8% in *ProCesA8-B:DCS_CURS2* lines.

To exclude the possibility that the observed shifts in cell wall composition were the consequence of the developmental delay in *ProCesA8-B:DCS_CURS2* lines, the cell wall characteristics of 87-day-old WT and four transgenic poplars with comparable heights (ranging from 118 to 127 cm and no shoot-tip necrosis) at the time of harvest were determined. These selected transgenic poplars still showed the previously described changes in cell wall composition (Supplementary Table 3).

### Wood of *ProCesA8-B:DCS_CURS2* Lines Has a Saccharification Potential That Is Equal to That of the WT

**FIGURE 5:**
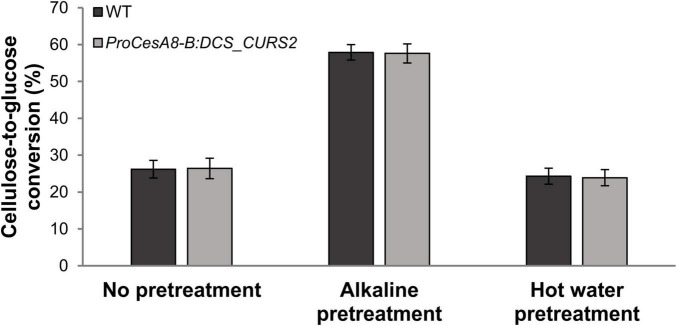
Saccharification potential of stem biomass from WT and *ProCesA8-B:DCS_CURS2* poplars. Cellulose-to-glucose conversion efficiency after 48 h of saccharification. Samples were saccharified using no pretreatment, alkaline pretreatment (62.5 mM NaOH, 3 h, 90°C) or hot water pretreatment (100% H_2_O, 3 h, 90°C). No significant differences between the WT and the transgenic lines were observed at the 0.01 significance level (Student’s *t*-test; WT, *n* = 8 biologically independent replicates; *ProCesA8-B:DCS_CUR*S2, *n* = 28 biologically independent lines). Error bars indicate standard deviation.

Curcumin-containing lignin in Arabidopsis is more easily cleaved under alkaline conditions, leading to an increase in saccharification potential (Oyarce et al., 2019). To test the impact of the structural changes in the lignin polymer of *ProCesA8-B:DCS_CURS2* stems, saccharification assays were performed on CWRs of 87-day-old WT and transgenic poplars after an alkaline pretreatment, with untreated and hot-water pretreated samples for comparison (Figure 5).

For all tested pretreatments, the cellulose-to-glucose conversion after 48 h of saccharification of the transgenic poplars was equal to that of the WT (Figure 5). The cellulose-to-glucose conversion without pretreatment was about 26%, after alkaline pretreatment about 57% and after hot water pretreatment about 24% both for WT and *ProCesA8-B:DCS_CURS2* samples.

## Discussion

### Translation of Fundamental Knowledge From Arabidopsis Into Poplar

Arabidopsis has been widely used as a model system for basic plant research and is a valuable model to study lignification (Meinke et al., 1998; Humphreys and Chapple, 2002; Koornneef and Meinke, 2010). The lignin biosynthetic pathways in Arabidopsis and the bioenergy crop poplar are similar, although poplar also incorporates additional units, like coniferyl and sinapyl *p*-hydroxybenzoates, into its lignin polymer (Lu et al., 2004, 2015; Morreel et al., 2004; Vanholme et al., 2019; Zhao et al., 2021; de Vries et al., 2022). Several lignin engineering strategies gave similar results in Arabidopsis and poplar. For example, greenhouse-grown *CCR-* or *CSE*-deficient Arabidopsis and poplar had lower amounts of lignin, higher saccharification efficiencies and yield penalties that depend on the level of residual target enzyme activity (Van Acker et al., 2013, 2014; Vanholme et al., 2013b; De Meester et al., 2020; de Vries et al., 2021a), whereas greenhouse-grown *4CL1*-deficient Arabidopsis and poplar had reduced lignin amounts and normal growth (Van Acker et al., 2013; Zhou et al., 2015). Introducing the curcumin biosynthetic pathway into Arabidopsis resulted in transgenic plants that produce and incorporate curcumin into their lignified cell walls while growing normally and having an up to 24% increased saccharification efficiency after alkaline pretreatment (Oyarce et al., 2019). To assess its value for applications, we evaluated this lignin engineering strategy in the bioenergy crop poplar. Introducing the curcumin biosynthetic pathway into poplar resulted in transgenic plants that also produce and incorporate curcumin into their lignified cell walls, but that exhibit altered growth phenotypes and no improvements in saccharification efficiency.

### *ProCesA8-B:DCS_CURS2* Poplars Produce and Incorporate Curcumin Into Their Lignified Cell Walls

In *ProCesA4:DCS_CURS2* Arabidopsis, phenylpentanoid- and curcumin-derived compounds were produced (Oyarce et al., 2019). These compounds, including phenylpentanoid monomers and dimers, free curcumin, and curcumin coupled to coniferyl alcohol, were also found here in *ProCesA8-B:DCS_CURS2* poplar. These findings suggest that, just like in Arabidopsis, (i) it is possible to engineer curcumin biosynthesis into poplar solely via the expression of *DCS* and *CURS2* under a secondary cell wall promoter, and (ii) curcumin is also capable of coupling with traditional monomers via radical-radical interactions during lignification in poplar.

As with *ProCesA4:DCS_CURS2* Arabidopsis (Oyarce et al., 2019), *ProCesA8-B:DCS_CURS2* poplar stem-cross sections showed a yellow coloration of their secondary-thickened cell walls under bright-field microscopy. As curcumin is a yellow pigment, this yellow color is indicative of the presence of curcumin (or a curcumin-derived compound) in the transgenic lines (Oyarce et al., 2019). Moreover, *ProCesA8-B:DCS_CURS2* poplar stem-cross sections also showed a strong fluorescence in their secondary-thickened cell walls under fluorescence microscopy (at an excitation wavelength of 488 nm). Because the fluorescence was similar to that seen after *in vitro* polymerization of curcumin in WT cross sections and as seen in *ProCesA4:DCS_CURS2* Arabidopsis (Oyarce et al., 2019), this confirmed the presence of curcumin in the secondary-thickened cell walls of the transgenic poplar lines. Finally, just like in *ProCesA4:DCS_CURS2* Arabidopsis (Oyarce et al., 2019), catalytic hydrogenolysis showed curcumin’s capability of translocating to and incorporating into the secondary cell wall of transgenic poplars.

### *ProCesA8-B:DCS_CURS2* Poplars Display a Significantly Altered Growth Phenotype

In contrast to *ProCesA4:DCS_CURS2* Arabidopsis that grew normally (Oyarce et al., 2019), *ProCesA8-B:DCS_CURS2* poplars suffered from yield penalties. At heights between 90 and 100 cm, the transgenic lines ceased growth, followed by desiccation and eventually necrosis of their shoot tips and the outgrowth of many new secondary shoots from the lower parts of the plant. The shoot-tip necrosis phenotype observed in *ProCesA8-B:DCS_CURS2* poplars clearly differed from the growth perturbations previously reported for lignin-modified plants (Chen and Dixon, 2007; Shadle et al., 2007; Coleman et al., 2008; Voelker et al., 2010; Ralph et al., 2012; Bonawitz and Chapple, 2013; Van Acker et al., 2013, 2014; Vanholme et al., 2013b). The latter, also coined lignin modification induced dwarfism (LMID), is typically associated with biomass and seed yield penalties. However, to the best of our knowledge, no shoot tip necrosis followed by the outgrowth of secondary branches was ever reported in lignin-modified plants. Additionally, the LMID observed in lignin-modified plants might be due to the loss of vessel cell wall integrity, leading to vascular collapse, which impedes water and nutrient transport from the roots to the aerial part of the plant (De Meester et al., 2018, 2021; Muro-Villanueva et al., 2019; Cao et al., 2020). Vascular collapse is often observed in growth-impeded lignin-engineered plants including poplars (Leplé et al., 2007; Coleman et al., 2008; Voelker et al., 2011; Cao et al., 2020; De Meester et al., 2021). However, *ProCesA8-B:DCS_CURS2* poplars deposited a higher fraction of lignin into their secondary cell walls and, similarly to *ProCesA4:DCS_CURS2* Arabidopsis (Oyarce et al., 2019), did not suffer from collapsed vessels indicating that the origin of their observed growth perturbations is not related to vascular collapse. Nonetheless, due to their underdeveloped root system, water (and nutrient) uptake might be limited in the transgenic poplars, leading to the observed growth phenotypes.

Although, to the best of our knowledge, not observed previously in lignin-modified plants, shoot tip necrosis followed by the development of secondary shoots is commonly observed in *in vitro* cultured plants and can be caused by a number of factors, including nutrient deficiency (calcium and boron), the concentration of cytokinins and nutrients in the medium, aeration, gelling agents, pH of the growth medium, and subculture period (Bairu et al., 2009; Srivastava and Joshi, 2013). Of all these causes, calcium and boron deficiencies are the most common causes of shoot tip necrosis in different plant species, including avocado, potato, chestnut, pear and, as here, poplar. Calcium deficiency in plants results in poor root growth (George et al., 2008), which is also observed in the *ProCesA8-B:DCS_CURS2* poplars. Furthermore, calcium starvation was shown to lead to a strong inhibition of shoot growth in poplar (Lautner et al., 2007). Also boron deficiency leads to inhibition of growth of shoot meristems as it is a structural constituent of cell walls (Hu and Brown, 1994; Apostol and Zwiazek, 2004). As curcumin can form a complex with boron (Hayes and Metcalfe, 1962) and was shown to regulate calcium-related processes and mobilization in human cell cultures (Kliem et al., 2012; Mayadevi et al., 2012), it is possible that the curcumin present in *ProCesA8-B:DCS_CURS2* poplars makes calcium and boron unavailable, causing deficiencies followed by shoot tip necrosis. Due to their underdeveloped root system, the uptake of calcium and boron from the soil in *ProCesA8-B:DCS_CURS2* poplars could additionally be compromised.

The severe reduction in the amount of *p*-hydroxybenzoates in *ProCesA8-B:DCS_CURS2* lignin (see also below) might also lead/contribute to the decreased health of the transgenic trees. To date, the biological function of lignin acylation remains unknown (de Vries et al., 2022). However, *phbmt1* poplars with nearly depleted *p*-hydroxybenzoates in stem lignin also display compromised early-stage growth in soil, although no shoot tip necrosis was observed (Zhao et al., 2021). When these poplars were grown for more than 3 months in soil, their growth appeared normal.

### Introducing the Curcumin Biosynthesis Pathway Into Poplar Significantly Alters Cell Wall Composition but Does Not Affect Saccharification Efficiency

Introducing the curcumin biosynthetic pathway into Arabidopsis and poplar redirects (part of) the feruloyl-CoA pool from the biosynthesis of the traditional monolignols toward curcumin biosynthesis. Nevertheless, the total lignin amount and H/G/S monomeric composition of *ProCesA4:DCS_CURS2* Arabidopsis remained unaltered, meaning that either the level of curcumin incorporation was minimal, or a feedback mechanism is in place to keep the total lignin amount at WT levels (Oyarce et al., 2019). In contrast to *ProCesA4:DCS_CURS2* Arabidopsis, *ProCesA8-B:DCS_CURS2* poplars had increased amounts of lignin, a decreased S/G ratio and an increased percentage of H units in the lignin when compared to the WT. These changes in lignin structure are most likely not a consequence of their aberrant growth, as they also occur in *ProCesA8-B:DCS_CURS2* poplars with a height comparable to that of the WT at the time of harvest. Hence, the structural changes to the lignin polymer were the consequence of the introduction of the *DCS* and *CURS2* transgenes leading to changes in the flux through the phenylpropanoid pathway and/or the possible activation of stress response pathways (i.e., the production of defense/stress lignin). Abiotic stresses typically induce the biosynthesis of H-rich lignin in both angiosperms and gymnosperms (Cesarino, 2019). Ozone-stressed poplar leaves were also shown to have higher lignin amounts, a lower S/G and a higher amount of H units incorporated into their lignin polymers (Cabane et al., 2004). A logical consequence of the relative increase in G and H units in the lignin of the transgenic lines is the observed relative increase in lignin 8-5 carbon-carbon linkages. Moreover, the relative decrease in S units in the lignin of the transgenic lines is in line with their relative decrease in *p*-hydroxybenzoates as, in poplar as well as in palms, it has been observed that *p-*hydroxybenzoates are almost exclusively bound to S units in the lignin of xylem fibers (Stewart et al., 2009; Lu et al., 2015; Regner et al., 2018; Goacher et al., 2021).

Although the cellulose amount of *ProCesA4:DCS_CURS2* Arabidopsis remained unaltered when compared to the WT control (Oyarce et al., 2019), that of *ProCesA8-B:DCS_CURS2* wood was significantly decreased. *ProCesA8-B:DCS_CURS2* wood also had increased amounts of matrix polysaccharides, which are mainly composed of hemicelluloses, when compared to the WT. As hemicelluloses might also covalently bind to lignin in trees (Terrett and Dupree, 2019), the observed changes in the cell wall composition of the transgenic lines might be the consequence of the activation of stress response pathways intended to produce lignin-carbohydrate complexes that strengthen the cell wall, as has been hypothesized for the ferulate-mediated cross-linking of polysaccharides with both polysaccharides and lignin in grasses (Monties, 1989; Bolwell, 1993; Ralph et al., 2004a; Ralph, 2010).

The cellulose-to-glucose conversion efficiency after saccharification using alkaline pretreatment was increased by up to 24% in *ProCesA4:DCS_CURS2* Arabidopsis (Oyarce et al., 2019), whereas that of *ProCesA8-B:DCS_CURS2* poplar was equal to that of the WT. The latter probably means that the effect of the incorporation of curcumin is nullified by other structural changes that occur in the secondary cell wall of the transgenic lines such as (i) their increased amount of lignin, which is the major limiting factor in the enzymatic hydrolysis of cell wall polysaccharides into simple sugars (Chen and Dixon, 2007; Van Acker et al., 2013; Vanholme et al., 2013b), (ii) the increased amount of 8-5 structures in their lignin that, unlike 8-*O*-4 ether linkages, resist harsh alkaline or acidic pretreatment conditions (Sarkanen and Ludwig, 1971), (iii) their increased amount of matrix polysaccharides and thus lignin-hemicellulose complexes that inhibit saccharification by enhancing cell wall recalcitrance (Yang and Wyman, 2004), and (iv) potentially, the reduced amount of *p*-hydroxybenzoates in their lignin; enhanced *p*-coumaroylation of poplar lignin leads to improved saccharification yields after alkaline pretreatment (Lapierre et al., 2021), suggesting that reduced *p*-hydroxybenzoylation of poplar lignin might have the opposite effect.

### Conclusion and Future Perspectives

Introducing *DCS* and *CURS*2 under the control of a secondary cell wall promoter results in the production of curcumin and subsequent incorporation of this alternative monomer into the secondary cell walls of Arabidopsis and poplar. However, in contrast to Arabidopsis, this lignin engineering strategy results in increased lignification, significantly altered lignin composition, growth perturbations and shoot tip necrosis when applied in poplar. These differences in (growth) phenotype display the importance of translational research in crops early during scientific development.

To generate transgenic poplars with cell walls more amenable to deconstruction without settling in yield, the proposed strategy might still be valuable if it can be fine-tuned to avoid the stress responses and growth defects. For example, using a different promoter to drive the expression of the curcumin biosynthesis genes (with a different spatial and/or temporal expression pattern) might lead to transgenic trees with more desirable characteristics. Indeed, here, the secondary cell-wall specific *CesA8-B* promoter was used that mainly confers expression in the xylem, but also has activity in phloem fibers and shoot tips (Joshi et al., 2004; Suzuki et al., 2006). As phloem fibers are mostly involved in providing support, biosynthesis of curcumin in these cells probably does not affect growth. However, the expression of the curcumin biosynthesis genes in the shoot tip, albeit to low levels, might contribute to the observed shoot tip necrosis. By restricting *DCS* and *CURS2* expression to the xylem, this adverse phenotype could potentially be avoided. To achieve this, a xylem-specific promoter is required to drive the curcumin biosynthesis genes. Examples include *ProDX15*, that confers expression in developing xylem cells (Jeon et al., 2016), *ProMX3*, that confers expression in mature xylem tissue (Nguyen et al., 2016), and *ProSNBE*, that confers expression in vessels (and sometimes also ray cells; De Meester et al., 2021). Alternatively, translation of this engineering strategy to other crops (e.g., maize) might lead to successful production and incorporation of curcumin into the lignified cell wall without affecting yield (just like in Arabidopsis).

## Data Availability Statement

The original contributions presented in this study are included in the article/Supplementary Material, further inquiries can be directed to the corresponding author.

## Author Contributions

BD, RuV, and WB designed the experiments. BD, PO, ReV, YT, TV, and JV performed the experiments. BD, PO, RuV, ReV, YT, TV, and JR collected and analyzed data. BD wrote the article with contributions from all authors. All authors contributed to the article and approved the submitted version.

## Conflict of Interest

The authors declare that the research was conducted in the absence of any commercial or financial relationships that could be construed as a potential conflict of interest.

## Publisher’s Note

All claims expressed in this article are solely those of the authors and do not necessarily represent those of their affiliated organizations, or those of the publisher, the editors and the reviewers. Any product that may be evaluated in this article, or claim that may be made by its manufacturer, is not guaranteed or endorsed by the publisher.
