# Supplementary material for: Engineering Curcumin Biosynthesis in Poplar Affects Lignification and Biomass Yield
Source: Front Plant Sci. 2022 Jul 4;13:943349. doi: 10.3389/fpls.2022.943349 (PMC9289561; doi:10.3389/fpls.2022.943349)
Supplement: Supplementary file 1 [file Data_Sheet_1.docx]

Supplementary Material

**Supplementary Table 1. Sequences used to generate the *ProCesA8-B:DCS_CURS2* construct.** For cloning of the *ProCesA8-B:DCS_CURS2* construct, we refer to Oyarce et al. (2019) and the Materials and Methods section.

| **>*ProCesA8-B* (*Populus trichocarpa*)**  GACACCGAATGCTTTACACAACCCACAATCATATAAAAAATTAATTCAGACATCAAGCAAGCCATAGGACATGCTAGCTAAAAATATAGACAGAAAAGGTTGTGTATACACGCAAAAACATACTCATCGAGGACAATACTTGCATCAGCAAGACATTAATTTGATAGTCGCATCCTTTGACAGAAGTTCTTGAAAGTCATCACAATGAATAAAAGTTGTCAATCCACTAGTAAAGAATTCTGAAACAAGAGCCTGTAAAATTTTTAAAATATATTCATTAGCAAAATGGAAAGTAACTAGGAAAAAAATATTGAATTAAGTTTCTTTGGCTATGTTATTCCCTCTTTCAAGAGATTTTGTTTTTGTTATTGTTGATTTTCTTCCGGCTTTCTACTCTCCTTTTTCTCAGGAATCAAAGACCTTGGTTTTTCCAGATCAAGGACCCTAGCCAATTTGCCTATAATAGGCCTCTAAAACCCGTTTCCCATGAAAGGAGAAAGCATAGGGAGAAAGAGAAAGTGAGCAAGCGTAGTGAGAAAAACAGAAAATTAGGAGAAAAAAAGAAGGAAGATGAGATAGAAAAGAGAAAGAAATAGACAGCAAAAAAGCACACAGGGAAGAAAAAAGATAAAGAAAAGATAGAAAGAAAATCGAGATATAAAAGAATAAAAAGGAAAAAAGAAGTTTTTTTAAGGTTGAGAAAAAAGTAAGGAAGAGAATCTTGAGATTTTGCAAGAAATAGGTTCCATTTGGACTACTCTGTGGGTGGCTATGTTGCTGCTGTGTCCTGTGTCACATTTTCAATGTTCGGGGAGAAGGGGAACCTGATAATTTCTGGTGGAAATGATAAGTTGATCAGTGAAAATGTGGGATTGCTCCAAATATCATGAAGCAGGGCAGACCGATGGTAACGAAGATGTTCTACGTCTTAACATTGATTTGAGCAAAAGGTCGATTTTTATTGTCTTTTGCTCGTGTTGGAATCCTTTCATCTCATACTTTCCATTTTGGCAGGTCAATTGGTTCTGTAAAACTCCAACTGATTCAGAAAACTTGTTGTTTGTGACGCAAAAAAAGTAGTAGAGGTCAATTCTGTTTCTTAGATTAGAGCTACAACATCATAGACTCCTGGAGAGCATGCCTTGATTCTGTTCAGGAGACGATAGTTTCCGGTTCGTTGAATGGCTTTGTTCACTTCTGGTCTAGCAATTTGCAAAAGAAGTTACAAAACAAATGCATATTATGTAAATTTAACAAGAGATGGGTTCTATAGTCACTTATTTATGCCCATAATTTGTTCTGGGGTTACTCTTTATAGTCTGATTCGAAGTTGCAAACTGCCGTTTCTGGTATTGCAATTATGTAGCCATAAACTGTTAATCCTGTTCCTATTAGTGGACCAACAACCAGATATATGGGCTCAGCGTCGTAAAAGAGATCTCCATTCTACGTTTCTTCCTATTTTTTCCGTTTCAGTGAGAGAATTACCCTGATACATTGATATGATGATTGATGATTATGGGAACCATGCCGATGTTAGACACTAGACCATCTGGATCCTGCCAGTTTTCTGTTCACATGGCATCCCAGCCCAAGATCATGTGTTTATACTCACTAATGACTTGTATTGAAAGTTTGTTAGTTGAAGATGTGCTCTGCCCAACAGAAACCTTCCTTAAATTTCCCGCAAATTTTTCAAAACTTGTCACTTACACCCCAAAAAATAGACGTTGCTTCTCACTTATGTTTCTCTGCAAAACACATGACACCAACTCCCCAACCGCCATACCCCACCAACCCACCACCCTCAACCTTCTCTTCGCCATTACAAAAATGTCAGTACCACCCTCTGAAAGACACCAACACACCCTAGCTTTGGTTAGGGTATTTGATATAAAAACAAGGCCAAAACAAAAGATTGGAAGGAAGCAGAGGAAGACCCTCTTGAAAGAATTGAAGAATTGGAGTTGTAAAGAGCTGGTAAAGTGGTAATAAGCAAG |
| --- |
| **>*DCS* (Turmeric, complete coding sequence, GenBank: AB495006.1)**  ATGGAAGCGAACGGCTACCGCATAACTCACAGCGCCGACGGGCCGGCGACGATCTTGGCCATCGGCACCGCCAACCCCACCAACGTCGTCGATCAGAACGCTTATCCCGACTTCTATTTCCGGGTCACCAACTCCGAGTATCTGCAGGAACTCAAAGCCAAGTTTAGGCGCATCTGTGAGAAAGCGGCCATCAGGAAGAGGCACTTGTACTTGACTGAGGAGATTTTGCGGGAGAATCCTAGCTTGCTGGCTCCCATGGCGCCGTCGTTCGACGCGCGGCAGGCGATCGTGGTGGAGGCGGTGCCGAAGCTGGCGAAGGAGGCGGCGGAGAAGGCGATCAAGGAGTGGGGCCGCCCCAAATCGGACATCACGCACCTCGTCTTCTGCTCCGCGAGCGGAATCGACATGCCCGGCTCCGACCTGCAGCTTCTCAAGCTGCTCGGGCTCCCGCCGAGCGTCAATCGCGTCATGCTCTACAACGTCGGGTGCCACGCCGGTGGCACCGCCCTCCGCGTCGCCAAGGACCTCGCGGAGAACAACCGCGGCGCGCGGGTGCTCGCCGTCTGCTCCGAGGTCACCGTGCTCTCCTACCGCGGCCCCCACCCCGCCCACATCGAGAGCCTCTTCGTCCAAGCTCTGTTTGGCGACGGCGCCGCCGCGCTCGTGGTCGGGTCCGACCCCGTCGATGGCGTCGAGCGCCCCATCTTCGAAATCGCCTCGGCATCCCAAGTGATGCTTCCGGAGAGCGCAGAGGCGGTGGGCGGCCACCTCCGCGAAATTGGGCTGACCTTCCACCTCAAGAGCCAGCTTCCGTCGATCATCGCGAGCAACATCGAGCAGAGCCTGACGACTGCGTGCTCGCCGCTGGGGCTGTCGGACTGGAACCAGCTGTTCTGGGCGGTTCACCCCGGCGGCCGAGCGATCCTGGACCAGGTGGAGGCGCGGCTCGGACTGGAGAAGGACCGGCTCGCCGCGACGCGGCACGTACTCAGCGAGTACGGCAACATGCAGAGCGCCACGGTGCTGTTCATCCTGGACGAGATGCGGAACCGCTCGGCTGCGGAGGGCCACGCCACCACCGGCGAGGGGCTCGACTGGGGCGTGCTGTTGGGCTTCGGCCCGGGACTCTCCATCGAGACCGTCGTCCTCCATAGTTGCAGACTGAACTAG |
| **>*CURS2* (Turmeric, complete coding sequence, GenBank: AB506762.1)**  ATGGCGATGATCAGCTTGCAGGCGATGCGCAAGGCGCAGAGAGCTCAAGGTCCGGCCACCATCTTGGCCGTCGGCACCGCCAACCCGCCCAATCTCTACGAGCAGGACACGTATCCCGACTACTACTTCCGCGTCACCAACTCCGAGCACAAGCAGGAGCTCAAGAACAAGTTCCGCCTCATGTGCGAGAAGACGATGGTGAAGAGGCGGTATCTTTACCTGACGCCGGAGATCCTGAAGGAGCGGCCGAAGCTGTGCTCGTACATGGAGCCGTCGTTCGACGACCGGCAGGACATCGTGGTGGAGGAGGTGCCGAAGCTGGCCGCGGAGGCAGCGGAGAACGCCATCAAGGAGTGGGGCGGCGACAAGTCCGCCATCACCCACCTGGTCTTCTGCTCCATCAGCGGCATCGACATGCCCGGAGCTGACTACCGCCTCGCCCAGCTCCTCGGACTCCCGCTCGCCGTCAACCGCCTGATGCTCTACAGCCAGGCCTGCCACATGGGCGCCGCCATGCTGCGCATAGCCAAGGACATCGCCGAGAACAACCGCTCCGCGCGCGTCCTCGTCGTCGCCTGCGAGATCACCGTGCTCAGCTTCCGCGGCCCGGACGAGCGCGACTTCCAGGCGCTGGCCGGCCAGGCCGGCTTCGGGGACGGCGCCGGCGCGATGATCGTCGGGGCCGACCCCGTCCTCGGCGTCGAGCGGCCGCTCTACCACATCATGTCGGCGACTCAGACGACGGTACCGGAGAGCGAGAAGGCGGTGGGGGGCCACCTCCGCGAGGTGGGGCTGACCTTCCACTTCTTCAACCAGCTGCCGGCGATCATCGCCGACAACGTGGGGAACAGCCTGGCGGAGGCGTTCGAACCGATCGGGATCAAGGACTGGAACAACATCTTCTGGGTGGCGCACCCGGGCAACTGGGCCATCATGGACGCCATCGAGACCAAGCTGGGCCTGGAACAGAGCAAGCTGGCCACCGCACGCCACGTCTTCTCCGAGTTCGGCAACATGCAGAGCGCCACCGTCTACTTCGTGATGGACGAGCTCAGGAAACGGTCGGCGGCGGAGAACCGGGCGACCACCGGCGACGGGCTCCGGTGGGGCGTGCTCTTCGGCTTCGGCCCGGGCATCAGCATCGAAACCGTCGTGCTCCAAAGCGTGCCGCTTTAG |
| **>*T2A***  GAGGGCAGAGGAAGTCTGCTAACATGCGGTGACGTCGAGGAGAATCCTGGCCCA |

**Supplementary Table 2. Height and growth rate of *ProCesA8-B:DCS_CURS2* and WT poplars grown in the greenhouse for 87 days.**

|  | **Height measurements (cm)** | | | | **Growth rate (cm/day)** | | |
| --- | --- | --- | --- | --- | --- | --- | --- |
| **Sample** | **Height day 57** | **Height day 60** | **Height day 80** | **Height day 87** | **Between day 57 - day 60** | **Between day 60 - day 80** | **Between day 80 - day 87** |
| **Wild type** | | | | |  |  |  |
| WT6 | 62 | 67 | 107 | 121 | 1.67 | 2.00 | 2.00 |
| WT7 | 60 | 69 | 108 | 122 | 3.00 | 1.95 | 2.00 |
| WT9 | 60 | 65 | 105 | 119 | 1.67 | 2.00 | 2.00 |
| WT11 | 62 | 68 | 112 | 127 | 2.00 | 2.20 | 2.14 |
| WT12 | 65 | 72 | 112 | 126 | 2.33 | 2.00 | 2.00 |
| WT13 | 63 | 68 | 112 | 127 | 1.67 | 2.20 | 2.14 |
| WT14 | 62 | 70 | 108 | 122 | 2.67 | 1.90 | 2.00 |
| WT15 | 58 | 66 | 104 | 118 | 2.67 | 1.90 | 2.00 |
| ***ProCesA8-B:DCS_CURS2*** | | | | |  |  |  |
| *ProCesA8-B:DCS_CURS2* line 1 | 62 | 67 | 103 | 107 | 1.67 | 1.80 | 0.57 |
| *ProCesA8-B:DCS_CURS2* line 2 | 66 | 69 | 100 | 101 | 1.00 | 1.55 | 0.14 |
| *ProCesA8-B:DCS_CURS2* line 3 | 56 | 60 | 71 | 72 | 1.33 | 0.55 | 0.14 |
| *ProCesA8-B:DCS_CURS2* line 4 | 70 | 73 | 104 | 105 | 1.00 | 1.55 | 0.14 |
| *ProCesA8-B:DCS_CURS2* line 5 | 54 | 60 | 92 | 94 | 2.00 | 1.60 | 0.29 |
| *ProCesA8-B:DCS_CURS2* line 6 | 61 | 66 | 97 | 97 | 1.67 | 1.55 | 0.00 |
| *ProCesA8-B:DCS_CURS2* line 7 | 60 | 62 | 62 | 63 | 0.67 | 0.00 | 0.14 |
| *ProCesA8-B:DCS_CURS2* line 8 | 58 | 60 | 72 | 73 | 0.67 | 0.60 | 0.14 |
| *ProCesA8-B:DCS_CURS2* line 9 | 76 | 79 | 105 | 105 | 1.00 | 1.30 | 0.00 |
| *ProCesA8-B:DCS_CURS2* line 10 | 72 | 77 | 100 | 100 | 1.67 | 1.15 | 0.00 |
| *ProCesA8-B:DCS_CURS2* line 11 | 65 | 70 | 74 | 74 | 1.67 | 0.20 | 0.00 |
| *ProCesA8-B:DCS_CURS2* line 12 | 42 | 47 | 56 | 56 | 1.67 | 0.45 | 0.00 |
| *ProCesA8-B:DCS_CURS2* line 13 | 54 | 60 | 90 | 92 | 2.00 | 1.50 | 0.29 |
| *ProCesA8-B:DCS_CURS2* line 14 | 70 | 74 | 102 | 102 | 1.33 | 1.40 | 0.00 |
| *ProCesA8-B:DCS_CURS2* line 15 | 68 | 71 | 98 | 98 | 1.00 | 1.35 | 0.00 |
| *ProCesA8-B:DCS_CURS2* line 16 | 39 | 43 | 90 | 90 | 1.33 | 2.35 | 0.00 |
| *ProCesA8-B:DCS_CURS2* line 17 | 64 | 71 | 93 | 97 | 2.33 | 1.10 | 0.57 |
| *ProCesA8-B:DCS_CURS2* line 18 | 67 | 73 | 90 | 90 | 2.00 | 0.85 | 0.00 |
| *ProCesA8-B:DCS_CURS2* line 19 | 66 | 68 | 98 | 107 | 0.67 | 1.50 | 1.29 |
| *ProCesA8-B:DCS_CURS2* line 20 | 47 | 50 | 62 | 62 | 1.00 | 0.60 | 0.00 |
| *ProCesA8-B:DCS_CURS2* line 21 | 43 | 49 | 81 | 90 | 2.00 | 1.60 | 1.29 |
| *ProCesA8-B:DCS_CURS2* line 22 | 66 | 72 | 101 | 106 | 2.00 | 1.45 | 0.71 |
| *ProCesA8-B:DCS_CURS2* line 23 | 36 | 44 | 77 | 90 | 2.67 | 1.65 | 1.86 |
| *ProCesA8-B:DCS_CURS2* line 24 | 65 | 67 | 95 | 95 | 0.67 | 1.40 | 0.00 |
| *ProCesA8-B:DCS_CURS2* line 25 | 65 | 73 | 112 | 118 | 2.67 | 1.95 | 0.86 |
| *ProCesA8-B:DCS_CURS2* line 26 | 72 | 80 | 113 | 121 | 2.67 | 1.65 | 1.14 |
| *ProCesA8-B:DCS_CURS2* line 27 | 68 | 75 | 115 | 126 | 2.33 | 2.00 | 1.57 |
| *ProCesA8-B:DCS_CURS2* line 28 | 64 | 72 | 108 | 119 | 2.67 | 1.80 | 1.57 |

**Supplementary Table 3. Cell wall characteristics of ‘tall’ *pCesA8-B:DCS_CURS2* poplars.** The cell wall residue (CWR) expressed as mass percentage of dry weight was determined gravimetrically after a sequential extraction. Crystalline cellulose content was determined by the Updegraff method and the mass loss during TFA extraction was used as an estimate of the amount of matrix polysaccharides. Lignin content was determined via the acetyl bromide (AcBr) method and expressed as mass percentage of CWR. Lignin composition was determined via 2D HSQC NMR. Differences between the WT and the transgenic lines were assessed with Student’s *t*-test (*0.01 < *P* < 0.05; ***P* < 0.01; WT, n = 8 biologically independent replicates; *ProCesA8-B:DCS_CUR*S2, n = 4 biologically independent lines). H, *p*-hydroxyphenyl; S, syringyl; G, guaiacyl; PB, *p*-hydroxybenzoate (see also Figure 4).

|  | **WT** | **Tall *ProCesA8-B:DCS_CURS2*** |
| --- | --- | --- |
| **CWR (% dry weight)** | 90.2 ± 2.8 | 79.9 ± 3.1** |
| **Cellulose (% CWR)** | 40.4 ± 6.2 | 35.3 ± 6.7 |
| **Matrix polysaccharides (% CWR)** | 40.8 ± 3.5 | 50.9 ± 4.2** |
| **Acetyl bromide lignin (% CWR)** | 15.8 ± 1.2 | 19.7 ± 1.7** |
| **NMR-derived aromatic units** |  |  |
| % H | 0.2 ± 0.1 | 2.3 ± 0.9** |
| % S | 66.1 ± 1.2 | 55.8 ± 4.1** |
| % G | 33.8 ± 1.2 | 41.9 ± 3.2** |
| S/G | 1.96 ± 0.10 | 1.35 ± 0.20** |
| % PB | 5.9 ± 0.9 | 0.3 + 0.2** |
| **NMR-derived interunit linkages** |  |  |
| % β-Aryl ether (8-*O*-4; **A**) | 89.1 ± 1.9 | 87.8 ± 0.9 |
| % Phenylcoumaran (8-5; **B**) | 1.8 ± 1.3 | 3.0 ± 0.5 |
| % Resinol (8-8; **C**) | 9.1 ± 0.7 | 9.3 ± 0.5 |


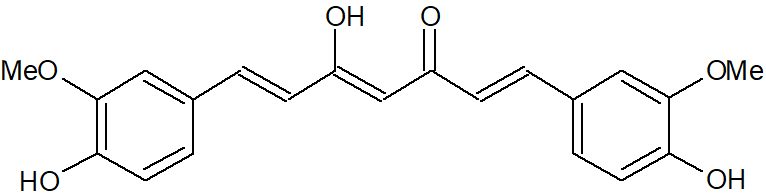

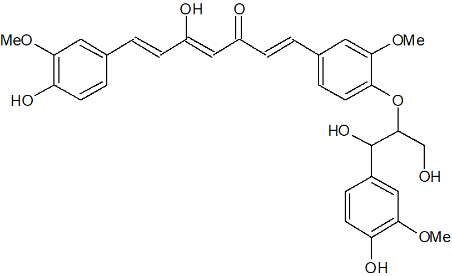

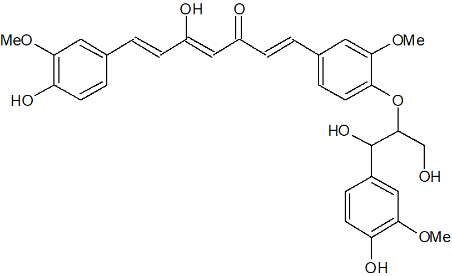
**
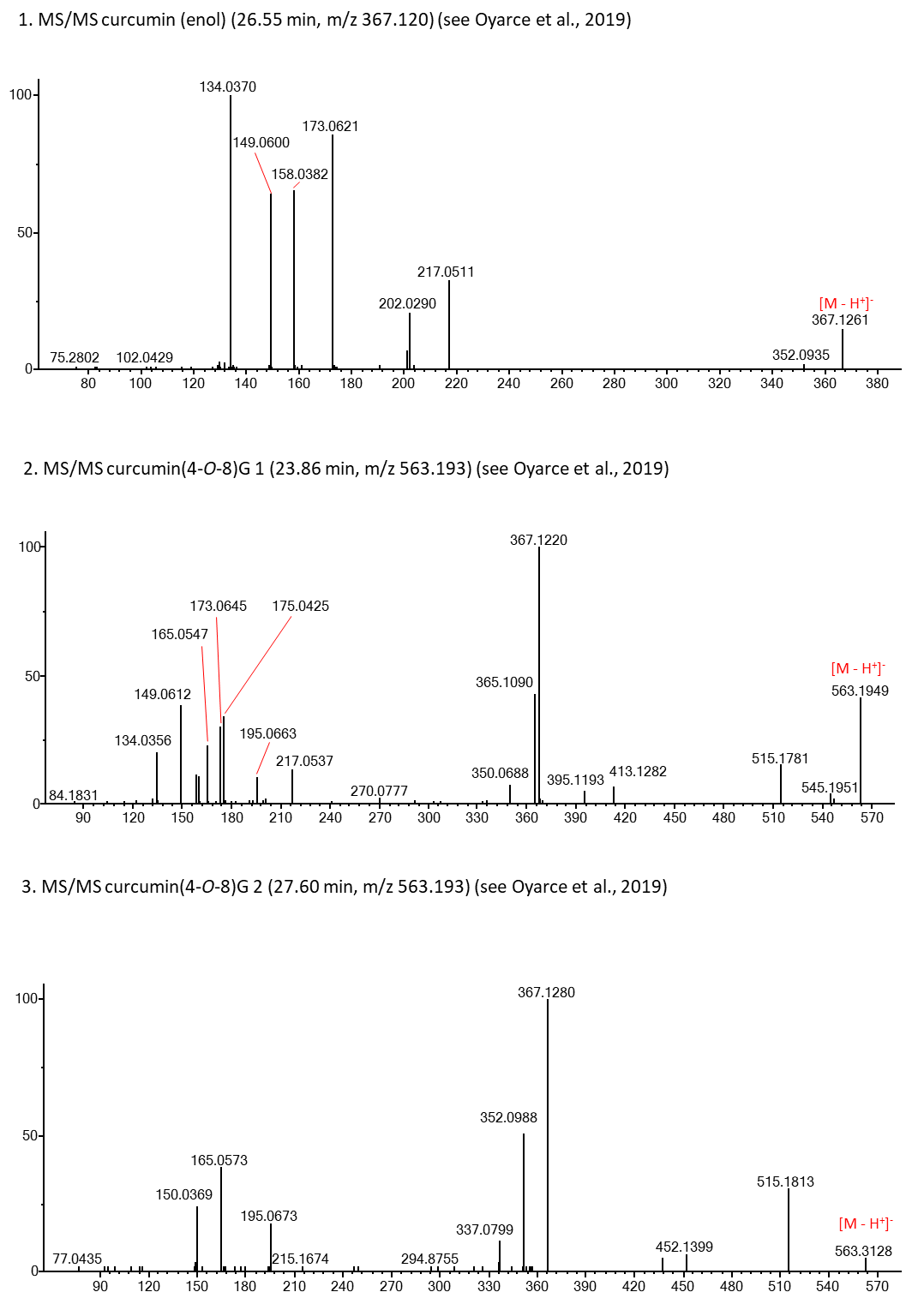
**

**
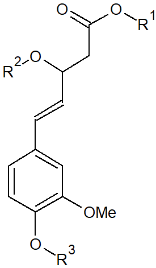
**
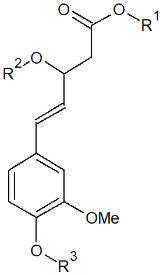
**
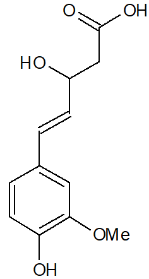

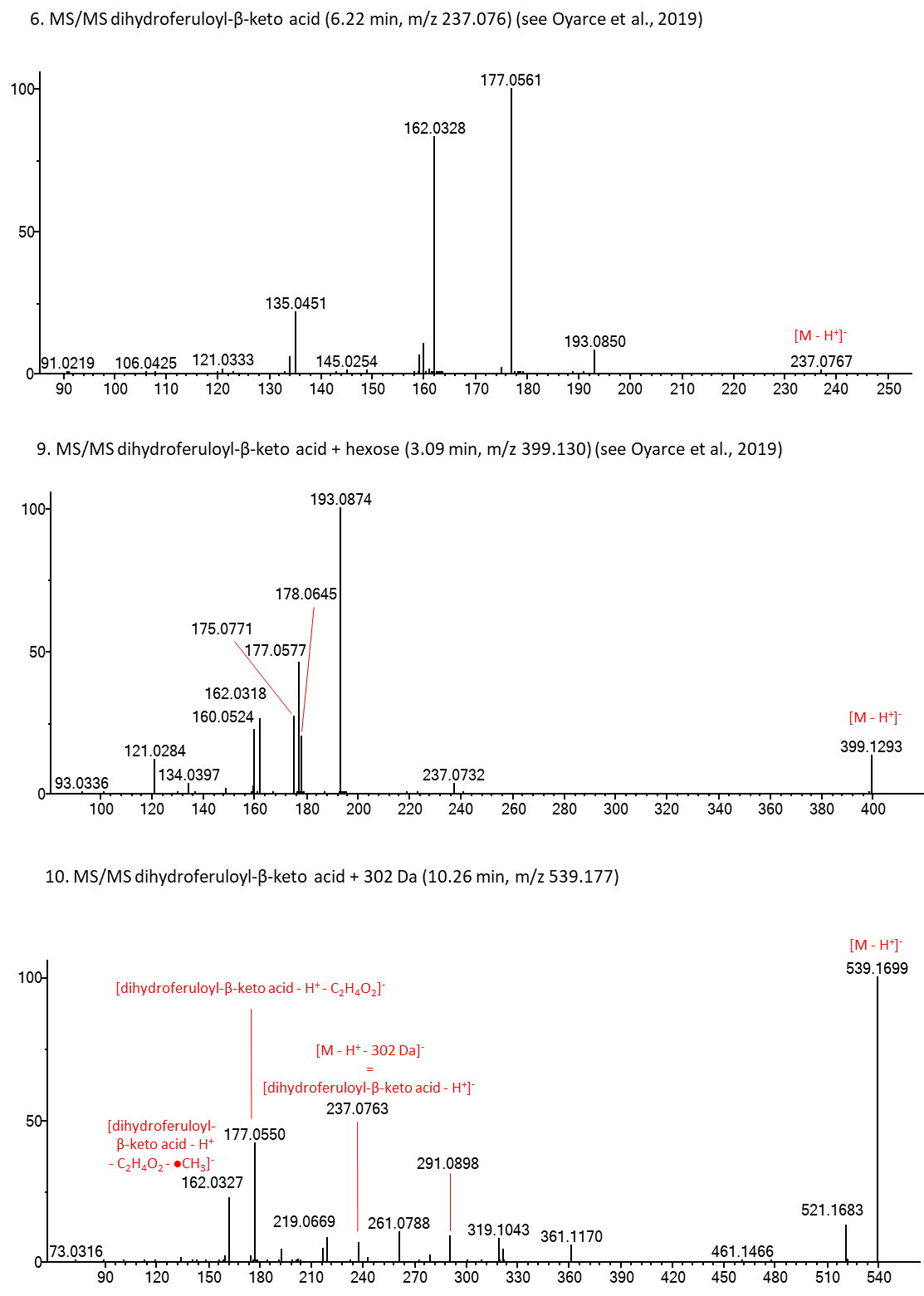
**

R^1^ = 302 Da & R^2^ = R^3^ = H

R^2^ = 302 Da & R^1^ = R^3^ = H

R^3^ = 302 Da & R^1^ = R^2^ = H

R^1^ = hexose & R^2^ = R^3^ = H

R^2^ = hexose & R^1^ = R^3^ = H

R^3^ = hexose & R^1^ = R^2^ = H


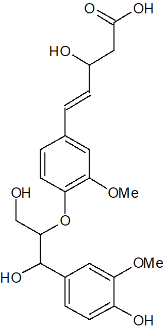

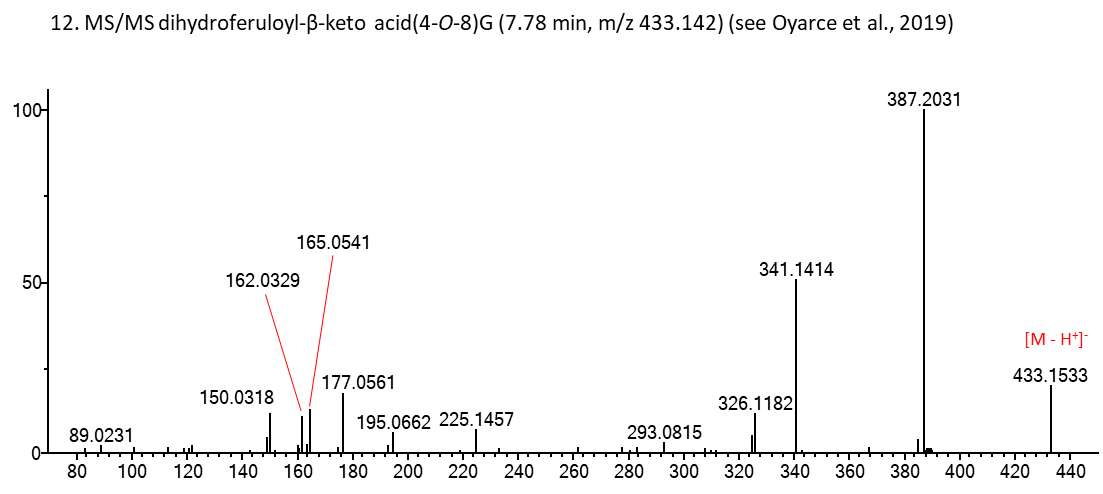


**Supplementary Figure 1. Phenolic profiling of *ProCesA8-B:DCS_CURS2* poplars.** MS/MS fragmentation spectra for compounds detected in xylem extracts of *ProCesA8-B:DCS_CURS2* plants. For each compound, a representative MS/MS spectrum is shown. The reasoning for the tentative structural identification is indicated on the spectra in red or found in Oyarce *et al.* (2019) as indicated. The numbers of the compounds correspond with those in Table 1.


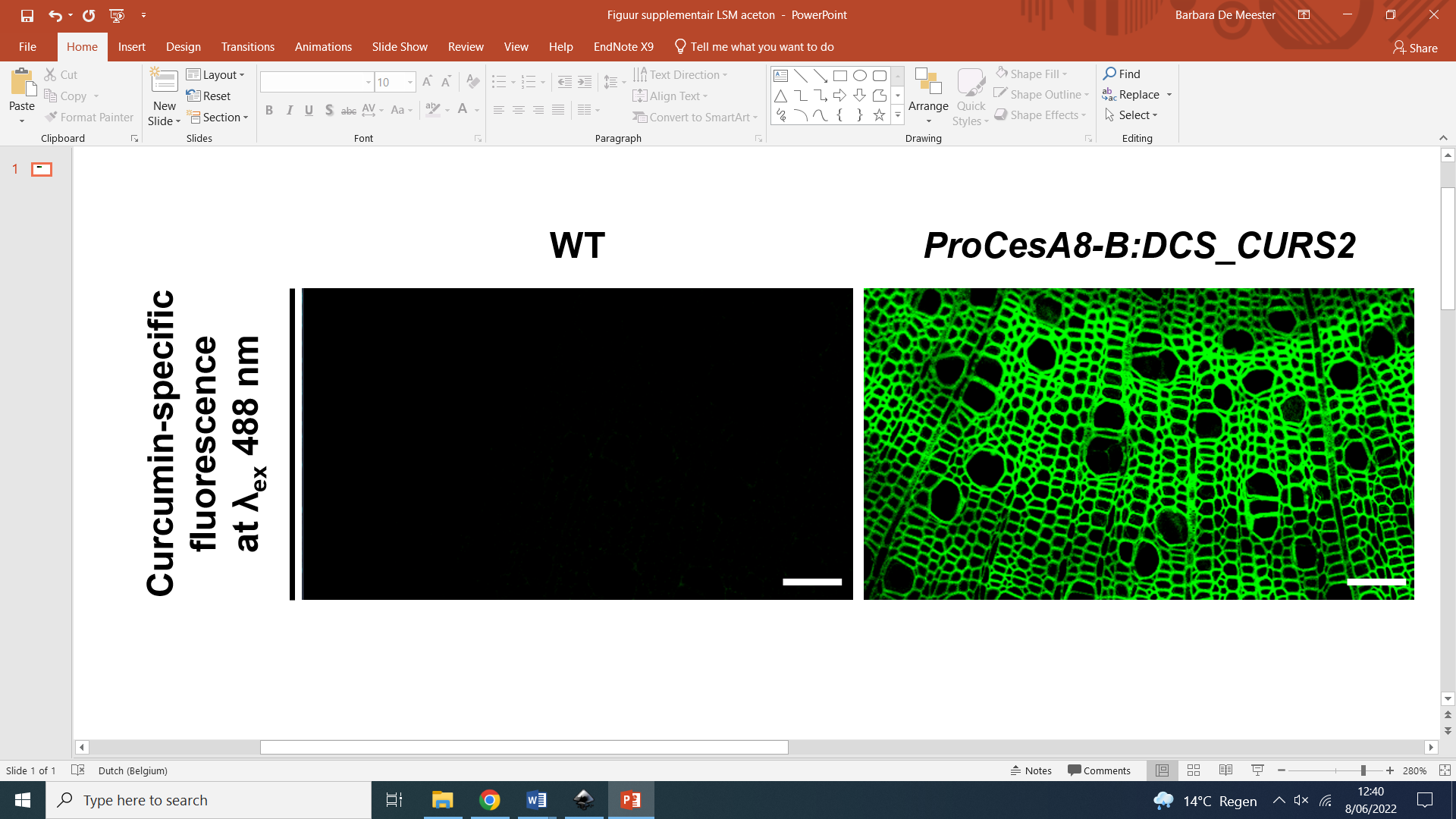


Supplementary Figure 2. Fluorescence microscopy on transverse stem sections of WT and *ProCesA8-B:DCS_CURS2* poplars incubated in acetone for 2 h. After removal of the soluble, non-covalently linked curcumin, the curcumin-specific fluorescence signal (excitation wavelength of 488 nm) was still observed i­n the cell wall of *ProCesA8-B:DCS_CURS2* poplars, while being absent in that of the WT. Scale bar: 100 µm.

**
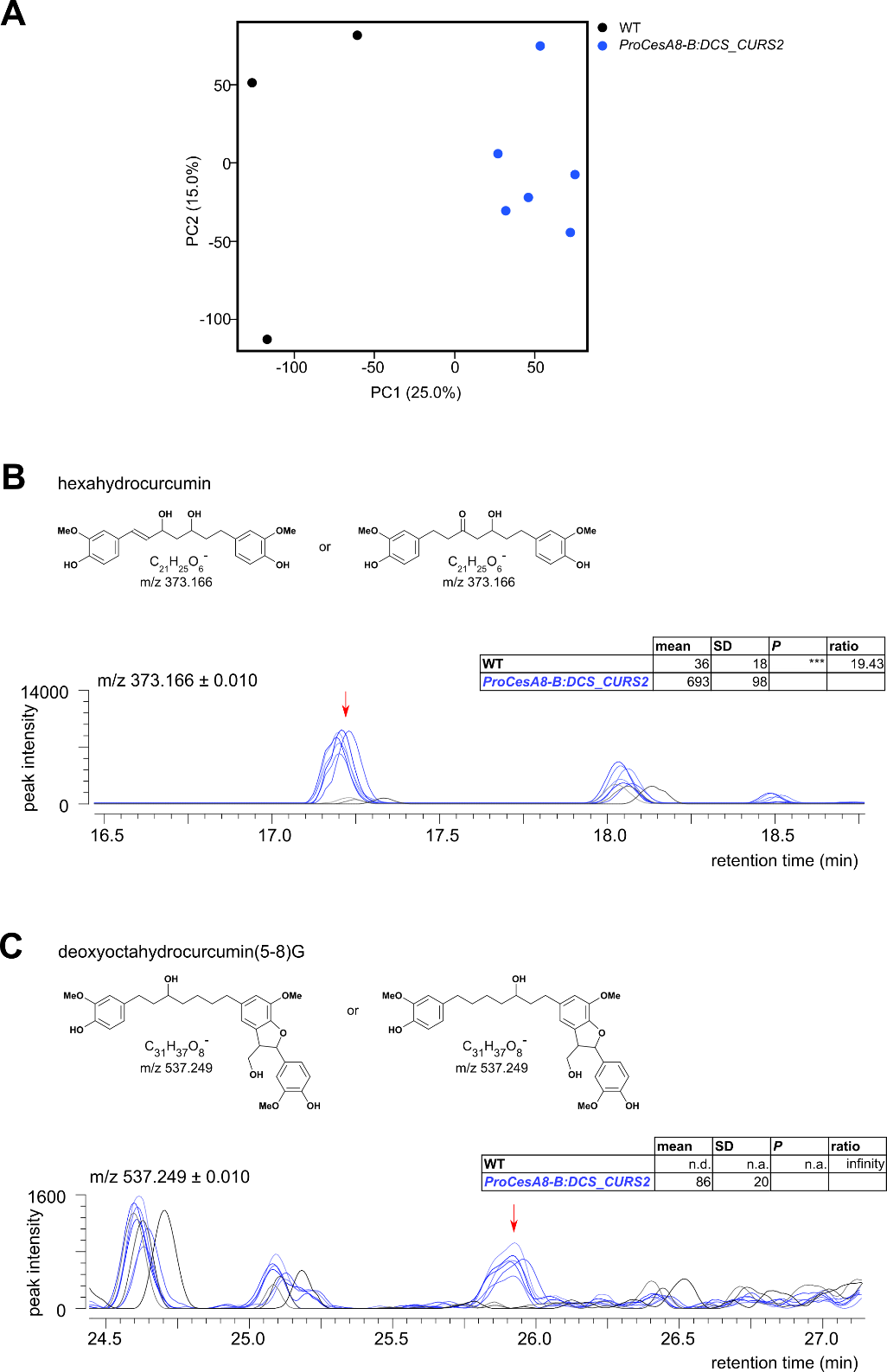
**

**Supplementary Figure 3. Characterization of catalytic hydrogenolysis lignin oil from *ProCesA8-B:DCS_CURS2* poplar stems.** (**A**) PCA showing the separation between UHPLC-MS profiles of *ProCesA8-B:DCS_CURS2* and WT lignin oils based on PC1. (**B-C**) Targeted search in the chromatograms of *ProCesA8-B:DCS_CURS2* (blue) and WT (black) lignin oils for *m/z* values that were derived from curcumin (coupling products). Two matches were found of which the intensities were significantly higher in *ProCesA8-B:DCS_CURS2* compared to WT: hexahydrocurcumin (**B**) and deoxyoctohydrocurcumin(5-8)G (**C**). These products were putatively structurally characterized based on high-resolution *m/z* (no MS/MS was available). Peak area (mean) ± standard deviation (SD) are expressed in counts. Differences between the WT and the transgenic lines were assessed with Student’s *t*-tests where applicable (****P* < 0.001; WT, n = 3 biologically independent replicates; *ProCesA8-B:DCS_CUR*S2, n = 6 biologically independent lines). Ratio: average *ProCesA8-B:DCS_CURS2* / average WT. n.d., not detected; n.a., not applicable. The low values for hexahydrocurcumin found in WT are probably the consequence of residual products from transgenic samples that remained in the reactor even after washing with acetone after each sample is processed.


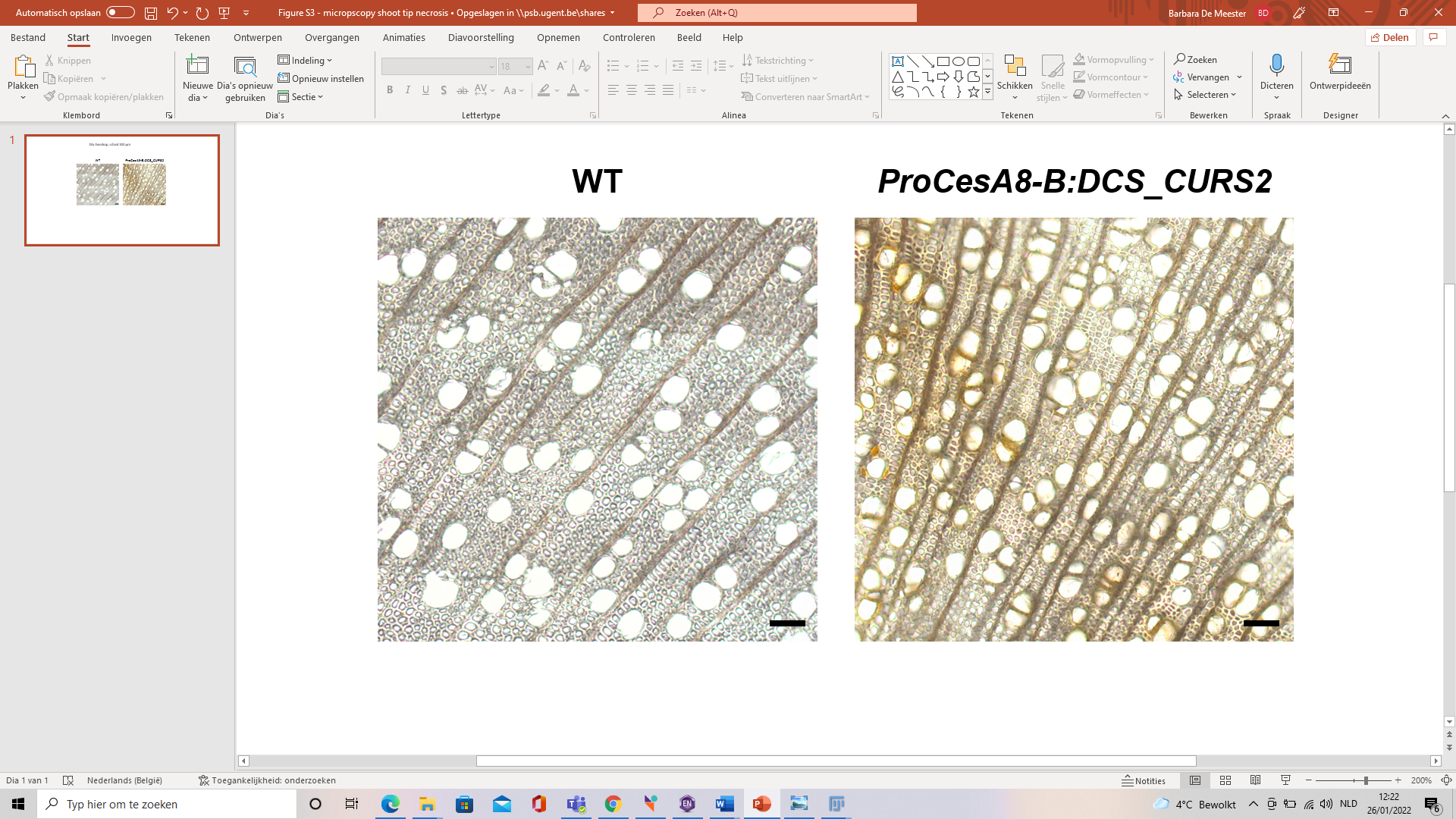


**Supplementary Figure 4. Bright-field microscopy on transverse stem sections of 4-month-old *ProCesA8-B:DCS_CURS2* poplars that developed shoot-tip necrosis**. Sections were imaged in water. A yellow coloration was observed in the cell walls of *ProCesA8-B:DCS_CURS2* lines, whereas the cell walls of the WT were gray. Both WT and transgenic lines displayed round, open vessels. Scale bars: 100 µm.
